# Placebo Analgesia Reduces Costly Prosocial Helping to Lower Another Person’s Pain

**DOI:** 10.1177/09567976221119727

**Published:** 2022-09-29

**Authors:** Helena Hartmann, Paul A. G. Forbes, Markus Rütgen, Claus Lamm

**Affiliations:** 1 Social, Cognitive and Affective Neuroscience Unit, Department of Cognition, Emotion, and Methods in Psychology, Faculty of Psychology, University of Vienna; 2 Social Brain Laboratory, Netherlands Institute for Neuroscience, Royal Netherlands Academy of Arts and Sciences

**Keywords:** empathy, prosocial behavior, decision-making, placebo analgesia, pain, effort, open data, preregistered

## Abstract

Painkiller administration lowers pain empathy, but whether this also reduces prosocial behavior is unknown. In this preregistered study, we investigated whether inducing analgesia through a placebo painkiller reduced effortful helping. When given the opportunity to reduce the pain of another person, individuals experiencing placebo analgesia (*n* = 45 adults from Austria; 21 male, 24 female) made fewer prosocial choices at the lowest helping level and exerted less physical effort when helping, compared with controls whose pain sensitivity was unaltered (*n* = 45; 21 male, 24 female). Self-reported empathic unpleasantness positively correlated with prosocial choices across the whole sample. While not replicating group differences in empathy, a mediation analysis revealed that the level of unpleasantness to other people’s pain fully mediated the effect of placebo analgesia on prosocial choices. Given the importance of prosociality for social cohesion, these findings have broad potential implications both for individuals under the influence of painkillers and for society at large.

The frequent use of opioid painkillers has been recognized as a global health concern, with negative effects on individuals and society (see Bolshakova et al., 2019, for a review). Pain medication has widespread effects on our own pain perception, but some studies show that it also impacts how we emotionally resonate with conspecifics (Mischkowski et al., 2016, 2019; see Ratner et al., 2018, for a review). Past research using a systemic placebo administration route (oral placebo pill) has causally linked an upregulation of the opioidergic system to reduced affective pain empathy, suggesting that the intake of painkillers may have detrimental effects on social motivation and subsequent behaviors (De Pascalis & Vecchio, 2022; Rütgen, Seidel, Silani, et al., 2015; Vecchio & de Pascalis, 2021). While the effects of psychopharmacological manipulations on firsthand pain and empathy for other people’s pain have been demonstrated, their potential downstream effects on prosociality remain to be established. Understanding these effects is fundamentally important, as these processes not only are key drivers of group bonding and social cohesion but also strongly contribute to individual and societal well-being (see De Waal, 2008, for a review; Rameson et al., 2012).

A series of experimental studies has shown that individuals whose firsthand pain was reduced by placebo analgesia (i.e., the belief that an inert treatment acts as a potent painkiller) showed reductions in self-reported pain empathy and decreased activation in key areas of the brain’s affective “pain empathy network” (Rütgen et al., 2021; Rütgen, Seidel, Riečanský, & Lamm, 2015; Rütgen, Seidel, Silani, et al., 2015; Vecchio & de Pascalis, 2021). Importantly, follow-up studies investigating whether these effects extend to the somatosensory system (using administration of a placebo gel to a selected body part rather than a pill affecting pain processing in a systemic fashion) did not find effects (Hartmann, Riva, et al., 2021; Hartmann, Rütgen et al., 2021). Of note here is that placebo mechanisms in firsthand pain (Petrovic et al., 2002; Zubieta et al., 2005; Zubieta & Stohler, 2009) and pain empathy (Rütgen et al., 2018, 2021; Rütgen, Seidel, Silani, et al., 2015) have been linked to an upregulation of the endogenous opioid system. Single-dose administration of acetaminophen (i.e., paracetamol, Tylenol), a non-opioidergic painkiller, also reduced self-reported empathy (Mischkowski et al., 2016, 2019). Thus, psychopharmacological manipulations of our own pain state causally and selectively influence how we share the affective-motivational component of others’ pain.

Empathy and prosociality are closely linked, with evidence both for somatosensory, cognitive, and affective domains (see Yin & Wang, 2022, for a meta-analysis). High trait and state empathy can act as powerful drivers of prosocial tendencies and behaviors (Brethel-Haurwitz et al., 2018; Crockett et al., 2014; Lockwood et al., 2014; Lockwood, Ang, et al., 2017; Lockwood, Hamonet, et al., 2017; Morelli et al., 2014; Story et al., 2020; see De Waal & Preston, 2017; Eisenberg et al., 2010; Eisenberg & Miller, 1987; Lamm et al., 2019, for reviews). Gallo et al. (2018) found that disrupting primary somatosensory cortex altered the coupling between pain perceived in another person and monetary donations to reduce that pain, Morelli et al. (2014) linked septal activity during empathy to daily helping, and Lockwood et al. (2014) identified emotion regulation as a moderator. On the other hand, it has been pointed out that more empathy may not always lead to more prosociality but that aspects connected to the former (such as personal distress) may also impair the latter (see Bloom, 2017; Decety & Cowell, 2014; Lamm & Majdandžić, 2015, for reviews).

However, whether altering firsthand pain sensitivity also impacts helping has not been systematically investigated. Answering this question will have major implications, as it could demonstrate that painkillers interfere not only with how we represent others’ emotions but also with how we behave towards others. The objective of this study was, thus, to investigate how reducing pain sensitivity through a well-established placebo analgesia manipulation influences helping. We preregistered the hypothesis that placebo analgesia would modulate prosocial behavior. This would be revealed by participants showing reduced willingness to choose an effortful helping option, longer reaction times (RTs) when making such choices, and a reduced amount of actual force exerted. However, we also considered the possibility that placebo analgesia could increase prosocial behavior in the preregistration (for further details regarding this hypothesis, see the preregistration).

**Statement of Relevance**

The increased and widespread use of opioids has been recognized as a major individual and public health threat, with a global average prevalence around 0.7% (28.6–30 million people). One underinvestigated aspect of this issue is how the intake of painkillers affects our social interactions. Previous studies highlighted detrimental effects of psychopharmacological interventions targeting the opioidergic system on empathic abilities. However, it is unclear whether reduced pain sensitivity also leads to changes in prosocial behavior. In this experimental behavioral study, we tested whether lowering pain sensitivity by means of placebo analgesia lowers helping behavior, measured by exerting physical effort to reduce another person’s pain. Placebo analgesia not only decreased participants’ willingness to invest effort but also decreased the actual effort they exerted when helping. These detrimental effects of analgesia on prosociality may have far-reaching implications not only for individuals under the influence of painkillers but also for social cohesion in societies in which analgesics are regularly consumed.

## Open Practices

As suggested by Simmons et al. (2012), we report how we determined our sample size, all data exclusions, all manipulations, and all measures in the study. This study was preregistered on the Open Science Framework (OSF) prior to data collection (see https://osf.io/g3acp/; deviations from the preregistration are listed in the Supplemental Material available online). All data and code to analyze the data and reproduce the figures are available on the OSF (https://osf.io/vcydf/).

## Method

### Procedure

#### Session overview

**Fig. 1.**
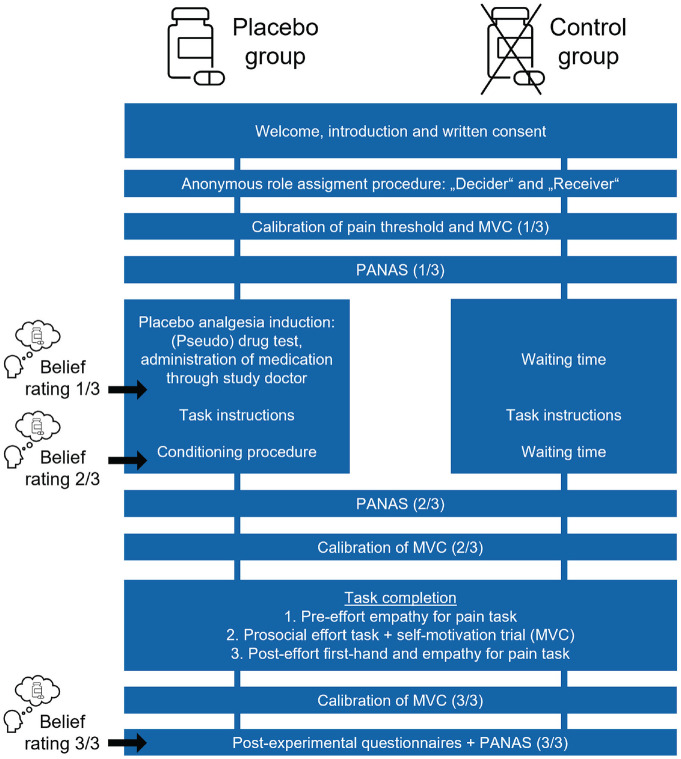
Overview of the experimental session for the placebo group and the control group. Real participants were always chosen as the decider. Participants in both groups underwent the same steps, except for the placebo analgesia induction. During this time, the control group had an equally long waiting time. Belief ratings about the effectiveness of the pill were collected only from the placebo group. PANAS = Positive and Negative Affect Schedule (Krohne et al., 1996); MVC = maximum voluntary contraction.

Participants took part in one behavioral session in pairs, with the other participant being a gender-matched confederate posing as the second participant (see Fig. 1 for an overview; see the Supplemental Material for more detailed procedures). All participants received the same financial compensation, and the ethics committee of the University of Vienna approved all procedures (Application No. 00412). Participants were explicitly told that they could discontinue their participation at any time without negative consequences and were debriefed about all deceptive elements upon conclusion of the study.

#### Role assignment and calibrations

The participant and confederate were asked to complete a “random role-assignment procedure” determining the roles of receiver and decider in the prosocial effort task, as in previous studies (Crockett et al., 2014; Lockwood, Hamonet, et al., 2017). The participant, who was always chosen as the decider, would make choices involving the other person receiving pain, and the confederate would always receive electrical stimulation. Anonymity (except for gender and name) was ensured over the whole session to limit the effects of reputation and reciprocity (as per Lockwood, Hamonet, et al., 2017, participants might act more prosocial just because they think they might meet the other person later again).

All subsequent steps relate to the real participant as the decider. Then, we employed a pain calibration as in Rütgen, Seidel, Silani, et al. (2015), where electrical stimulation was given to the dorsum of the left, nondominant hand to gain average stimulation intensities between 0 (*not perceivable*) and 8 (*extremely painful*) using a Digitimer DS5 Isolated Bipolar Constant Current Stimulator (Digitimer Ltd., Fort Lauderdale, FL, USA). Calculated intensities for the average ratings of 1 (*perceivable but not painful*) and 4 (*medium painful*) were later used for the conditioning, whereas 1 and 7 (*very painful*) were used for the pain task. After this, effort was calibrated using maximum voluntary contraction (MVC), whereby participants were asked to grip a handheld dynamometer (Vernier, Beaverton, OR) in their right, dominant hand with as much force as possible. This ensured that the effort levels used in the task were relative to each participants’ individual strength. The maximum of four MVC trials (two before and two after the placebo induction) was taken to calculate the effort levels (30%–70% of that value) in the prosocial effort task. The effort calibration was completed three times (before and after the placebo induction and at the end of the session). To assess changes in motivation and fatigue over time, we also measured each participant’s MVC twice in a row at the same three time points as the NASA Task Load Index ratings measuring effort, physical demand, and unpleasantness of the effort exertion (Hart & Staveland, 1988; see Supplemental Material).

#### Placebo analgesia induction

Next, the placebo group underwent a placebo analgesia induction using an inert pill presented as an “effective and powerful painkiller” as part of verbal suggestions by a medical student acting as the study’s medical doctor. After a waiting time of 10 min for the pill “to take effect,” classical conditioning was performed to amplify the placebo effect. Participants received stimulation with a medium intensity (rated as 4 in the pain calibration), coupled with corresponding feedback suggesting that the pill had substantially reduced pain (e.g., “This stimulus was rated as very/extremely painful before”). To measure placebo responding over the course of the session, we collected three belief ratings about the effectiveness of the “medication.” The control group did not undergo any such manipulations but had equally long waiting times to keep the overall session length the same. We additionally collected positive and negative mood ratings from both groups at three times using the Positive and Negative Affect Schedule (PANAS; Krohne et al., 1996).

#### Prosocial effort task

A major challenge in measuring prosocial behavior is that many existing paradigms involve only one-shot tasks, such as picking up dropped pens or donating part of a monetary endowment (see Thielmann et al., 2020, 2021 for overviews). These tasks may not capture moment-by-moment decisions necessary in everyday life. Moreover, they largely rely on self-report, which is prone to social desirability and often measures hypothetical intentions rather than actual behavior (see Martí-Vilar et al., 2019; Pfattheicher et al., 2022, for reviews).

To overcome these limitations, recently developed experimental paradigms require participants to invest physical effort over multiple trials (see Cameron et al., 2022; Contreras-Huerta et al., 2020, for reviews). These tasks show that individuals vary their helping on the basis of how much effort they need to put in and how rewarding that helping is (Lockwood, Hamonet, et al., 2017; Lockwood et al., 2021). Furthermore, participants’ decision-making in the form of choice behavior has been increasingly used in addition to subjective indicators such as ratings to more comprehensively measure the full extent of prosocial behavior (Crockett et al., 2014; Lockwood et al., 2016; Story et al., 2020).

**Fig. 2.**
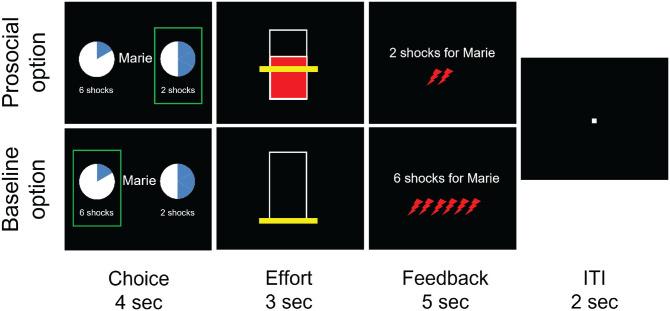
Timeline of the prosocial effort task. As the decider, the real participant had to choose between a baseline rest option (no effort exertion) and a more effortful prosocial option: In the latter case, participants were asked to exert 30%, 40%, 50%, 60%, or 70% of their maximum voluntary contraction (MVC; the more filled the circle, the greater the effort level; 1/6th was filled as the baseline option, and each additional slice represented a higher effort level). One of these MVC percentages that participants exerted was combined with the possibility to reduce the number of shocks the other participant was going to receive in that trial by one, two, three, four, or five, respectively (indicated below the circle). This led to 5 × 5 = 25 possible combinations, each of which was presented three times. After the choice, participants immediately exerted their chosen effort while receiving real-time visual feedback to show how much effort they were exerting in comparison with the effort they had to reach (indicated by a yellow line, which had to be reached for at least 1 s out of 3 s). After the 3 s had elapsed, they received feedback on the shocks the other participant (allegedly) got. The feedback included text and red lightning shapes, which appeared one after the other to reinforce the belief that shocks were given to the receiver in real time. All information given on screen in the task was in German.

We therefore developed a paradigm to test whether placebo analgesia affects the effort individuals exert to reduce another’s pain. The prosocial effort task was adapted from Lockwood, Hamonet, et al. (2017) and Crockett et al. (2014): On every trial, participants were asked to make a choice between a baseline “rest” offer (no effort necessary), where the confederate received a fixed amount of six shocks, and a “work” offer involving exertion of physical effort (30%, 40%, 50%, 60%, or 70% of their MVC) to help the other participant (reducing the number of shocks the confederate would receive by one, two, three, four, or five shocks; Fig. 2). Every work-offer combination (5 effort levels × 5 shock reduction levels = 25 levels) was presented 3 times, leading to 75 choices. If the participants chose to help but failed to reach the chosen effort level, or when they did not respond within 3 s, the confederate received 10 shocks (see the Supplemental Material for more information on these “fail trials,” which did not differ between groups; all *p*s > .055).

The participants were told that the other participant would receive shocks they had rated as very painful (i.e., in the upper range of the rating scale) in their pain calibration. In fact, the receiver never received pain in this task. This deception was used to create a believable and realistic social situation in which decisions of the participants would ostensibly have real and live consequences for another person, to induce a conflict between costly investing of effort and helping to reduce another’s pain.

At the end of the prosocial effort task, MVC was measured again, but this time it was incentivized to determine participants’ motivation to earn a financial bonus for themselves. We used this one-trial measure as a control to assess whether the placebo influenced self-related motivation. Of note, while other studies have used a more closely matched self-related control (Contreras-Huerta et al., 2020 for review), we opted against this for practical and theoretical constraints of our study—balancing length, duration, believability, other-focus in terms of resource, and possible carryover effects between experimental and control conditions. As per our preregistration, if placebo analgesia acted exclusively on prosocial motivation, we would expect no group differences on this task. If, on the other hand, placebo analgesia modulates motivation to exert effort in general, we would expect a group difference.

#### Firsthand-pain and empathy-for-pain task

**Fig. 3.**
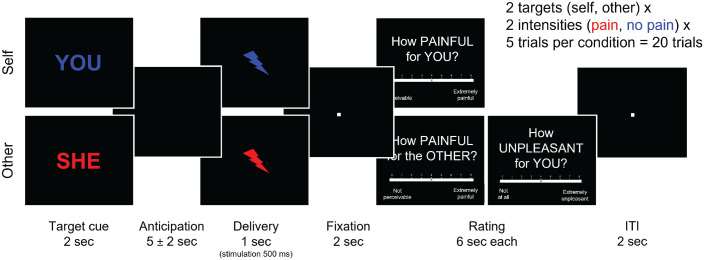
Timeline of the firsthand-pain and empathy-for-pain task. Participants completed measures of empathy for pain before and after the prosocial effort task. After the prosocial effort task, participants either received electrical stimulation themselves (self) or observed the second participant seemingly receiving such stimulation (other). Afterward, participants were asked to rate the pain intensity and unpleasantness of these stimulations. Before the prosocial effort task, participants supplied only empathy ratings and did not receive firsthand stimulation.

To evaluate analgesic effects on firsthand pain and empathy for other people’s pain, we asked participants to rate painful and nonpainful electrical stimulation (rated as 1, *noticeable but not painful*, or 7, *very painful*, in the pain calibration task) that either they received themselves (self trials) or the confederate received (other trials; Fig. 3; Rütgen, Seidel, Riečanský, & Lamm, 2015). Participants completed a short pre-effort empathy-for-pain task (two pain and two no-pain trials) before the prosocial effort task (as per the “other” condition in Fig. 3) and the full, posteffort firsthand-pain and empathy-for-pain task afterward. This structure was purposefully chosen to avoid firsthand pain experience before the prosocial effort task and to avoid counteracting any effects by delaying the effort task. This also allowed us to assess transfer effects of placebo analgesia to empathy for pain before (pre-effort) and after the effort task (posteffort), at two different time points in the session.

#### Postexperimental questions

At the end of the session, we assessed any doubts regarding the cover story (whether participants believed their anonymity was kept, whether their decisions were kept secret from the other participant, and whether they felt their decisions were monitored by the experimenters) and the placebo manipulation to identify nonresponders (see the Participants section in the Supplemental Material). We also measured how painful they remembered the average painful and nonpainful stimulation being in the pain task.

### Participants

Participants were German-speaking, young, right-handed university students with normal or corrected-to-normal vision who reported no history of neurological or psychiatric disorders. Our final sample size met our preregistered goal of 90 participants (45 participants per group)—age: *M* = 23.56 years, *SD* = 2.90 (placebo group) and *M* = 24.00 years, *SD* = 4.32 (control group). We based our group sizes on previous studies using tasks involving either effort or pain, as our task was a combination of the two (Crockett et al., 2014; Lockwood, Hamonet, et al., 2017). All exclusion criteria were defined a priori in the preregistration. Placebo analgesia nonresponders were determined as in Rütgen, Seidel, Silani, et al. (2015; see the Supplemental Material for more information): First, we recorded verbally expressed doubts about the analgesic effects of the pill or the cover story. Second, we analyzed differences of the belief scores about the effectiveness of the placebo before and after the induction procedure. Third, we took into account the number of conditioning trials needed to suggest pain relief. Dropouts and exclusions in each group were replaced until the group sizes of 45 were reached. These exclusion criteria were not related in any way to our two outcomes of interest, empathy for pain and prosocial behavior, but served to establish a reliable firsthand placebo analgesia effect as a prerequisite for the group comparisons. The two final groups were comparable regarding age and trait measures of sociocognitive and emotional abilities (see Table S1 in the Supplemental Material).

### Data collection and analysis

Tasks were implemented in Cogent 2000 (Version 1.33) running in MATLAB (Version R2018a; The MathWorks, Natick, MA). Data were processed and analyzed using MATLAB R2016a and RStudio (Version 4.1.0; R Core Team, 2020). All *p* values were interpreted two-sided. Wherever Mauchly’s test for sphericity was violated, we report *p* values using Greenhouse-Geisser correction.

### Manipulation checks

We conducted five manipulation checks, some of which we preregistered and others we added after data collection because of the novelty of the design. First, we evaluated the correct performance of the prosocial effort task (Manipulation Check 1) and the believability of the cover story in both groups (Manipulation Check 2; see the Supplemental Material).

Then we tested the effectiveness of the placebo analgesia induction procedure. The placebo groups’ belief in the effectiveness of the medication (Manipulation Check 3) was analyzed by calculating three paired-samples *t* tests comparing two of the three time points (before conditioning, after conditioning, after the session). Although these analyses were preregistered as exploratory, they were also employed in our previous studies using a very similar placebo analgesia induction with a gel instead of a pill (Hartmann, Riva, et al., 2021; Hartmann, Rütgen et al., 2021). On the basis of these studies, we expected a strong belief in the medication and an increased belief from before to after conditioning (see the Supplemental Material for exact criteria). We then aimed to evaluate whether the placebo analgesia induction influenced (a) firsthand pain as well as (b) other-related pain intensity and unpleasantness ratings (Manipulation Check 4); our goal was to replicate the placebo analgesia effect reported by Rütgen, Seidel, Silani, et al. (2015). As in that study, we expected placebo analgesia to downregulate participants’ firsthand pain as well as their pain empathy. We thus preregistered the hypothesis that placebo analgesia would lead to lowered empathic responses. We calculated two analyses of variance (ANOVAs) using pre-effort empathy ratings, with other pain intensity or unpleasantness ratings as the dependent variables, and the independent variables group (placebo, control) and intensity (pain, no pain). We then calculated an additional two ANOVAs using the posteffort firsthand-pain and empathy-for-pain ratings: The first ANOVA compared the self- and other-related pain-intensity ratings on the independent variables group (placebo, control), target (self, other), and intensity (pain, no pain); the second ANOVA compared the unpleasantness ratings on group (placebo, control) and intensity (pain, no pain). Analyses of pre-effort empathy for pain were not preregistered but mirrored the preregistered posteffort analyses.

To measure whether the placebo effect lasted until the end of the session (Manipulation Check 5), we compared the postexperimental rating about how much pain was felt on average in the pain task using a *t* test with the index of pain ratings (pain – no pain) as the dependent variable and group (placebo, control) as a between-subjects factor. This check was not preregistered but was also employed in our previous studies (Hartmann, Riva, et al., 2021; Hartmann, Rütgen et al., 2021).

### Preregistered core analyses

We calculated a mixed ANOVA with the proportion of work offers as the dependent variable and the factors effort level (1–5), shock reduction/helping (1–5), and group (placebo, control) as independent variables. We then repeated this analysis with RT as the dependent variable. As preregistered, both of these analyses were repeated using linear mixed models (LMMs) to detect more subtle differences using single-trial data, adopting a multiverse analysis approach (e.g., see Silberzahn et al., 2018, for behavior or Botvinik-Nezer et al., 2020 for imaging analyses). Last, we ran an LMM with area under the curve (AUC) of the force data as the dependent variable and group, effort level, shock reduction, and their interactions as fixed effects. We also included a subject-level random intercept. To check whether placebo analgesia influenced general motivation, we ran a *t* test to compare the two groups on the MVC and exerted force in the one-shot self-trial.

### Post hoc analyses

To evaluate the effects of the placebo induction on participants’ general strength and mood, we ran three ANOVAs with the dependent variables MVC, positive mood, and negative mood, using time (before induction, after induction, after the session) and group (placebo, control) as factors in all three analyses. We also investigated correlations between the two tasks (i.e., empathy for pain and prosocial behavior). To this end, we correlated the firsthand and other-related pain intensity/unpleasantness ratings in the firsthand-pain and empathy-for-pain task with the proportion of prosocial choices in the prosocial effort task. Moreover, we explored associations between prosocial choices and RT in the prosocial effort task.

Next, individuals differ in how much they value other people’s rewards relative to their own (prosocial/altruistic vs. individualistic oriented). We explored the relationship between prosocial choices in the prosocial effort task and social value orientation (SVO) by correlating the proportion of prosocial choices to each participants’ social value angle from the SVO by Murphy et al. (2011), which was calculated as the arc tangent of the ratio for other- versus self-related payoffs as an angle in degrees (with higher values meaning higher prosocial/other-related orientation). These correlations were not preregistered and are therefore exploratory and reported uncorrected for multiple comparisons.

Last, we calculated an exploratory mediation analysis to investigate the role of affect sharing in mediating the effect of placebo analgesia on prosocial choices. We operationalized the placebo effect as the independent variable by subtracting the firsthand-pain rating from the value 7 (i.e., the rating corresponding to the pain intensity delivered, as determined during prior individual pain calibration). The mediator variable consisted of the other-related unpleasantness rating in response to painful stimuli, and the dependent variable was the proportion of prosocial choices.

## Results

### Both groups showed correct task performance and cover story belief as well as similar strength, motivation, and mood

There were no significant group differences regarding correct performance of the prosocial effort task and believability of the cover story (Manipulation Checks 1 and 2; see Fig. S1 and Tables S2–S4 in the Supplemental Material for details). We also did not observe any group or time differences in participants’ general strength measured as MVCs over the course of the session (all *p*s > .561; see Fig. S2 and Table S5 in the Supplemental Material). In the analysis of participants’ effort exertion when aiming to win an additional monetary bonus for themselves, we observed no group differences in participant’s MVC (*p* = .432) or in the AUC of the exerted force (*p* = .175; in the Supplemental Material, see Fig. S3a for MVC data and Fig. S3b for force data). Evaluating participants’ mood at three time points, we observed that both positive and negative mood decreased significantly over the course of the session (both *p*s < .001), but these effects were similar for both groups (see Fig. S4a for positive mood, Fig. S4b for negative mood, and Tables S6 and S7 in the Supplemental Material).

### Placebo versus control group differed in firsthand pain but not in other-related pain intensity or unpleasantness ratings

**Fig. 4.**
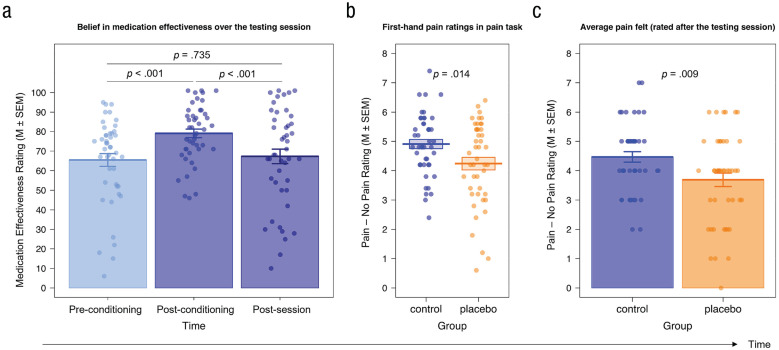
Manipulation Checks 3, 4, and 5, evaluating the firsthand placebo analgesia effect. (a) We observed high belief ratings in general and a significant increase in the belief in the effectiveness of the medication from before to after the conditioning procedure. Although this belief significantly decreased toward the end of the session, it did not drop lower than the initial belief before the conditioning. (b) In the firsthand-pain and empathy-for-pain task, participants in the placebo group rated the electrical stimulation they received on the back of their hand as significantly less painful than the control group. (c) Participants in the placebo group indicated at the end of the session that they remembered the stimulations in the firsthand-pain and empathy-for-pain task to be significantly less painful than participants in the control group. Dots represent individual data.

When assessing the placebo analgesia effect, we observed a significant increase in the belief about the effectiveness of the pill in the placebo group from before to after conditioning, *t*(44) = 3.92, *p* < .001, mean difference (*M*_diff_) = 13.58, 95% confidence interval (CI) = [6.59, 20.57], Cohen’s *d*_*z*_ = 0.58; before conditioning: *M* = 65.44, *SEM* = 3.24; after conditioning: *M* = 79.02, *SEM* = 2.21 (Manipulation Check 3; Fig. 4a). From after conditioning to after the session (*M* = 67.27, *SEM* = 3.71), belief ratings decreased significantly, *t*(44) = −4.05, *p* < .001, *M*_diff_ = −11.76, 95% CI = [-17.60, -5.91], Cohen’s *d*_*z*_ = 0.60, whereas postsession ratings did not differ significantly from the ones measured before conditioning, *t*(44) = 0.34, *p* = .735, *M*_diff_ = 1.82, 95% CI = [-8.97, 12.62], Cohen’s *d*_*z*_ = 0.05. Importantly, the average belief rating (*M* = 70.58, *SEM* = 2.06 on a scale from 0 to 100) indicated a generally high belief in the medication’s effectiveness.

**Fig. 5.**
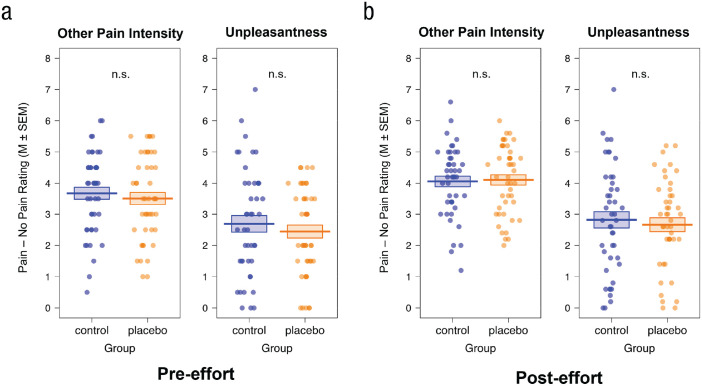
Manipulation Check 4, evaluating the effect of placebo analgesia on other-related pain intensity and unpleasantness ratings. Participants completed (a) pre-effort empathy-for-pain task (see Tables S8 and S9 in the Supplemental Material) and (b) the full posteffort firsthand-pain and empathy-for-pain task (see Tables S10 and S11 in the Supplemental Material). They rated pain intensity as well as their own unpleasantness when the other participant (allegedly) received electrical stimulation. We observed no effect of placebo analgesia on other pain intensity or unpleasantness in the pre- or posteffort data. Dots represent individual data.

Next, we analyzed the pre- and posteffort ratings in our firsthand-pain and empathy-for-pain task (Manipulation Check 4; Fig. 4b for firsthand pain, Fig. 5 for other-related pain intensity and unpleasantness; see the Supplemental Material for more detailed results). Analyses confirmed a significant placebo effect for firsthand pain, as shown by generally lower ratings in the placebo compared with the control group (main effect of group, *p* = .008) independent of intensity or target as well as a bigger rating difference in the index (pain – no pain stimulation) between self- and other-related stimulation—that is, control (self_pain – no pain_ – other_pain – no pain_) > placebo (self_pain – no pain_ – other_pain – no pain_)—for the control compared with the placebo group (Group × Intensity × Target interaction, *p* = .020) but no group differences regarding other-related pain intensity or unpleasantness ratings (*p*s > .128) in the pre- or posteffort pain-task data. Although the hypothesized group differences in other-related pain intensity and unpleasantness ratings went in the expected direction (placebo < control), they were not significant, and their effect sizes (Cohen’s *d*s between 0.04 and 0.15) were much smaller compared with those in previous studies that also used a between-subjects design and very similar setup (e.g., Rütgen, Seidel, Silani, et al., 2015, Cohen’s *d*s of firsthand analgesia effect between 0.62 and 0.79; Cohen’s *d*s of empathy analgesia effect between 0.44 and 0.76).

We further observed a significant difference between the average index for firsthand ratings (pain – no pain) in the task when participants were asked to remember the pain in that task at the end of the session, *t*(83.12) = 2.68, *p* = .009, 95% CI = [-0.20, 1.36], Cohen’s *d* = 0.57; Manipulation Check 5; Fig. 4c). In this analysis, the placebo group (*M* = 3.69, *SEM* = 0.23) remembered the self-directed electrical stimulation as significantly less painful than the control group (*M* = 4.47, *SEM* = 0.18).

Taken together, Manipulation Checks 3 to 5 showed the success of the placebo analgesia induction. Replicating previous studies, our results demonstrated that placebo analgesia downregulated firsthand-pain ratings in the placebo compared with the control group, and this effect lasted until the end of the session and thus beyond the part in which the prosocial effort task was performed. Contrary to previous research and our preregistered hypothesis, results showed that other-related pain intensity and unpleasantness ratings did not seem to be affected by the placebo analgesia manipulation, either when measured directly after the placebo analgesia manipulation or in the ratings given after the prosocial effort part of the experiment.

### Placebo group displayed reduced prosocial behavior compared with the control group in their choices and exerted force

**Fig. 6.**
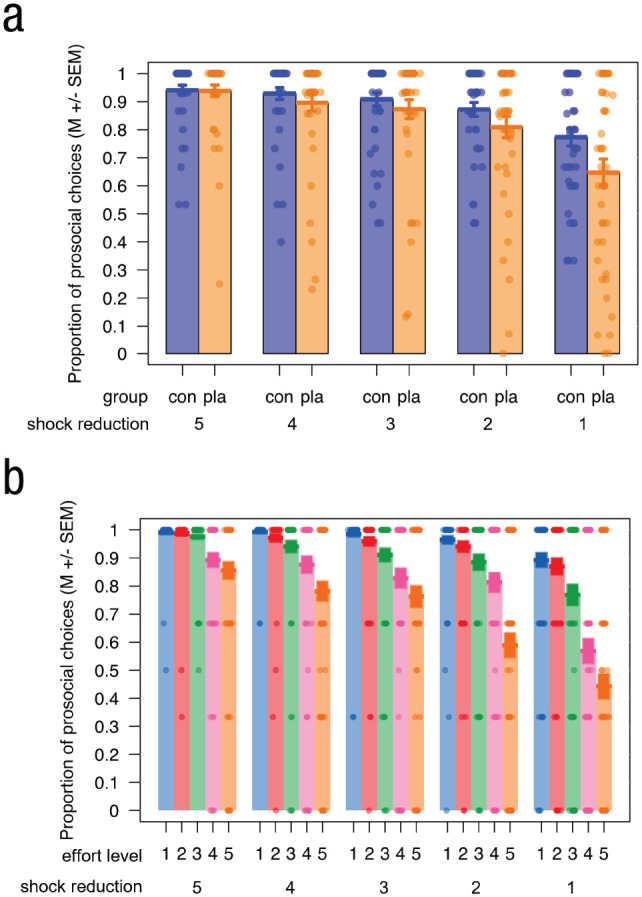
Main results in the prosocial effort task. (a) Participants in the placebo group (orange) chose the prosocial option significantly less often than the control group (blue) when the effect of helping was lowest (i.e., a reduction of only one shock), whereas the groups did not differ for the other shock reduction amounts (reduction of more than one shock; Group × Shock Reduction interaction). (b) Participants differentiated their helping behavior less over the five effort levels when they could help more compared with less (Effort Level × Shock Reduction interaction). Dots represent individual data.

The ANOVA analyzing the proportion of work offers chosen (Fig. 6; see Table S12 in the Supplemental Material) revealed main effects of effort level and shock reduction (see the Supplemental Material for further details). Both of these effects were expected from previous studies and predicted by our preregistered, directional hypotheses, whereas any interactions were left exploratory. Intriguingly, we observed a Group × Shock Reduction interaction, *F*(4, 352) = 4.10, *p* = .020 Greenhouse-Geisser corrected, $\hat{\eta }$ _*G*_^2^ = .01, showing that the placebo group displayed reduced prosocial behavior compared with the control group, dependent on the number of shocks the other would receive but independent of effort level (Fig. 6a). Post hoc tests confirmed that this group difference was significant only for a shock reduction of 1, that is, the lowest helping level (*p* < .001, value adjusted using Tukey). Last, we observed an Effort Level × Shock Reduction interaction, *F*(16, 1408) = 13.11, *p* < .001 Greenhouse-Geisser corrected, $\hat{\eta }$ _*G*_^2^ = .03, whereby the differences in the proportion of prosocial choices between the five effort levels increased with decreasing possibility to help, independent of group (Fig. 6b). In other words, participants differentiated their helping behavior less at the effort levels when they could help more compared with less. No other effects were significant. The generalized LMM of the same data largely mirrored the ANOVA results (see Table S13 in the Supplemental Material).

The same ANOVA analyzing RT during the choice phase revealed faster RTs for choices involving lower effort exertion as well as higher possibility to help over both groups (see Table S14 in the Supplemental Material). In other words, participants were faster in their decisions when they needed to put in less effort and could help more. Furthermore, we observed an Effort Level × Shock Reduction interaction, whereby the differences in RTs between the five effort levels decreased with increasing possibility to help, independent of group (all *p*s < .001). Regarding our preregistered hypotheses, no other effects were significant, showing no group differences in the choice RTs. The LMM of the same data mirrored the ANOVA results (see Table S15 in the Supplemental Material).

In the LMM analyzing the exerted force (see Table S16 in the Supplemental Material), we found a main effect of group, χ^2^(1) = 5.21, *p* = .022, whereby the placebo group (*M* = 0.43, *SEM* = 0.002) exerted less force than the control group (*M* = 0.44, *SEM* = 0.002) after having chosen to put in effort to help the other. Moreover, we again observed a main effect of effort, χ^2^(4) = 3,844.73, *p* < .001, showing that participants exerted more force with increasing effort level. We additionally found an Effort × Shock Reduction interaction, χ^2^(16) = 77.39, *p* < .001.

### Prosocial behavior correlated with choice RTs and unpleasantness but not pain intensity when participants observed others’ pain

We observed a significant positive association between the amount of prosocial choices and unpleasantness ratings across all participants (Spearman’s ρ = .22, *p* = .035 uncorrected), whereby higher levels of unpleasantness were associated with increased prosocial behavior (choosing the prosocial option more often; Fig. S5a in the Supplemental Material). We did not find a significant association between prosocial behavior and firsthand pain (ρ = .03, *p* = .768 uncorrected) or other pain-intensity ratings (ρ = .01, *p* = .920 uncorrected; Fig. S5b in the Supplemental Material). Moreover, we found a significant negative correlation between participants’ RTs when making their choice and the proportion of prosocial choices in the prosocial effort task (ρ = −.58, *p* < .001 uncorrected), showing that faster RTs were associated with increased prosociality and vice versa (see Fig. S5c in the Supplemental Material). Last, there was a significant positive correlation between participants’ trait SVO and their prosocial behavior in the prosocial effort task (ρ = .23, *p* = .028 uncorrected), showing that participants who described themselves as more other-oriented also had a higher proportion of prosocial choices, or vice versa (see Fig. S5d in the Supplemental Material).

### Affect sharing mediated the effect of placebo analgesia on prosocial choices

As previous studies found downregulating effects of placebo analgesia on affect sharing, we aimed to show that the effect of placebo analgesia on prosocial behavior in our study was mediated by the level of affect sharing using an exploratory mediation analysis. This showed that the effect of placebo analgesia on prosocial choices was fully mediated via the level of affect sharing in response to others’ pain (see Fig. S6 in the Supplemental Material). While the (average) direct effect (DE/ADE) between the placebo response and proportion of prosocial choices was not significant and reduced from *b_DE_* = −0.02 (*p* = .181) to *b_ADE_* = −0.01 (*p* = .686), the regression coefficient between affect sharing and prosocial choices was significant (*b* = 0.04, *p* = .043), and so was the effect of placebo responses on affect sharing (*b* = −0.37, *p* < .001). The indirect effect (average causal mediation effect [ACME]) was *b* = (.04*−.37) = −.01. We tested the significance of this indirect effect using bootstrapping procedures and computed the 95% CI by determining the indirect effects at the 2.5th and 97.5th percentiles. Standardized indirect effects were computed for each of 1,000 bootstrapped samples in the R package *mediation* (Tingley et al., 2014). The bootstrapped standardized ACME was *b* = −0.01, 95% CI = [−0.03, 0.00]. Thus, the indirect effect, which was the main effect of interest in our mediation analysis, was significant (*p* = .032).

## Discussion

We investigated the causal effects of a placebo painkiller on prosociality. Participants under placebo analgesia showed reduced willingness to put in effort to help others at the lowest helping level and exerted less force when putting in effort to help.

Our design revealed that placebo participants’ drop in helping behavior scaled with the amount of shock reductions: While it most strongly differed in the lowest shock condition, it was virtually identical for maximum shock reduction. This pattern seems counterintuitive, as one might expect effects to be strongest in more intense situations, but could be explained in terms of saliency and social norms: If there is high need for and effectiveness of helping, the incentive to help may be so high that placebo analgesia is not able to modulate behavior; conversely, if stakes are lower, placebo analgesia has leverage to exert its effects. In other words, while high-shock-situations are very salient and produce a strong (moral) urge to help without question, low-shock situations are more ambiguous and may prompt a more open-ended decision. This is in line with prior work by Gallo et al. (2018), who showed that participants donate more money on trials in which a confederate expresses more pain. Furthermore, Fehr and Fischbacher (2004) posit that prosocial norms depend on the expected beliefs and desires of the recipient, which, according to our results, may be perceived differently in high- versus low-pain conditions. As per the norm-activation model (Steg & de Groot, 2010), participants might be more aware of adverse consequences of their behavior in exactly those high-pain conditions, feel a stronger moral obligation, and thus prevent them more readily. This interpretation is supported by our RT data, in which all participants were quicker to help in high-shock-reduction situations. However, this hypothesis will have to be systematically tested in future studies. Even when choosing to help, the placebo group exerted less energy compared with controls (this effect was found only in the LMM and not the ANOVA). Lockwood, Hamonet, et al. (2017) reported similar findings when participants exerted effort for others compared with themselves. Our results suggest that placebo analgesia could exacerbate this “prosocial apathy.”

Although placebo analgesia successfully reduced firsthand pain, we did not replicate previous studies of reduced other-related pain intensity and unpleasantness (De Pascalis & Vecchio, 2022; Mischkowski et al., 2016; Rütgen, Seidel, Riečanský, & Lamm, 2015; Rütgen, Seidel, Silani, et al., 2015; Vecchio & De Pascalis, 2021). One explanation could be that our placebo induction was not strong enough to affect pain empathy. What speaks against this is that we found no group differences in empathy for pain measured directly after the induction but firsthand placebo analgesia effects lasting until the end of the session. Furthermore, there were several differences between our study and previous studies reporting placebo or painkiller effects on empathy: For example, task length, anonymity of participants toward each other, varying study doctors between sessions, and framing (the focus being on the prosocial effort task here and on the pain task previously), all of which could have influenced the way placebo analgesia affected pain empathy on a group level. Importantly, when considering individual differences in placebo responses and affect sharing in an exploratory mediation analysis, we found not only that the two were significantly related but also that affect sharing fully mediated the effects of placebo responding on prosocial choices. Our results thus highlight the importance of individual differences, as well as that altering firsthand pain may additionally influence prosociality via routes beyond empathy and affect sharing. What routes those are and how differences in perception (“what we feel”) and placebo responsiveness are linked to actions (“what we do”) are important avenues for further research.

Unpleasantness ratings when observing other people in pain are usually seen as a marker of affect sharing, while pain-intensity ratings are thought to measure cognitive-evaluative components of empathy (see Lamm & Majdandžić, 2015, for a review). Interestingly, exploratory analyses showed that the proportion of prosocial choices was positively associated with higher unpleasantness ratings when observing others in pain but not with self- or other-related pain-intensity ratings. Our post hoc mediation analysis extended this finding by showing that the effects of individual differences in placebo analgesia on prosocial choices were fully mediated by the level of affect sharing in response to other people’s pain. This resonates well with prior work linking affect sharing, empathic concern, and prosocial behavior (see Eisenberg & Fabes, 1990, for a review; Lengersdorff et al., 2020; Lockwood et al., 2016): Hein et al. (2010) found that self-reported trait empathic concern for another person’s suffering predicted later costly helping (enduring physical pain to reduce the other’s pain). Gallo et al. (2018), however, stressed a connection between cognitive-evaluative ratings of the pain intensity observed in others and subsequent donation behavior to reduce their pain. Taken together, the multifaceted nature of these findings shows that certain aspects of empathy-related responses may influence different aspects of prosociality (exerting effort, enduring pain, donating money). It also reminds us that empathy is not the only driver of prosocial behavior (see Bloom, 2017; Decety & Cowell, 2014; Lamm & Majdandžić, 2015, for reviews). To tease apart these various influences, future studies should find different ways to induce and measure facets of empathy and prosociality.

A higher proportion of prosocial choices was associated with faster RTs. This is in line with studies associating quicker decisions to greater prosociality (Chen & Krajbich, 2018; Rand et al., 2012). The proportion of prosocial choices also correlated with increased prosocial tendencies in trait SVO. This nicely links prosocial traits people ascribe themselves with their actual behavior, complementing studies demonstrating that the level of cooperativeness in SVO relates to contributing more hours for a prosocial cause (McClintock & Allison, 1989) and greater donations (Lange et al., 2010).

The present study had several strengths and limitations. We preregistered our study, clearly distinguishing confirmatory from exploratory findings and reducing the risk of Type I errors (Nosek et al., 2018). We aimed to exclude alternative explanations such as social norms, reciprocity, or reputation, as participants never met face to face (Crockett et al., 2014). Belief in our cover story highlighted that participants’ behavior was likely not influenced by these concerns. Anonymity may still have reduced empathic responses, through mechanisms such as objectification (Eklund, 2006), dehumanization (Cameron et al., 2015), or distancing (Cameron et al., 2019; Story et al., 2020). However, such effects would likely be independent of the placebo manipulation. As in Lockwood et al. (2021), we operationalized prosocial behavior using costly choices and exertion of physical effort and kept this behavior independent of monetary gains, group allocation, or study time. This setup investigated both people’s explicit choice behavior as well as the implicit energization of their actions, disentangling “true” from “superficial” helping behaviors. With this, we aimed to measure prosocial behavior in an ecologically more valid way, which may better translate to real-world prosocial acts. Nevertheless, future research should translate our laboratory-based findings to real-life measures of prosociality (e.g., using ecological momentary assessment or diaries; Morelli et al., 2014; Rameson et al., 2012). And although the present study provides insights into effects of altered pain sensitivity on social emotions and behavior, generalizations to people affected by pain-related disorders and frequent painkiller use will require different study designs and participants.

In sum, placebo analgesia decreased people’s willingness to exert effort to reduce others’ pain as well as their actual effort. Downregulation of one’s own pain sensitivity not only changes how we experience pain but also affects our decisions to help others. Notwithstanding independent validation and extension to more ecological settings, and especially to use of painkillers, this study has important implications for the social interactions of people under the influence of analgesics and with chronic pain conditions.

## Supplemental Material

sj-docx-1-pss-10.1177_09567976221119727 – Supplemental material for Placebo Analgesia Reduces Costly Prosocial Helping to Lower Another Person’s Pain Supplemental material, sj-docx-1-pss-10.1177_09567976221119727 for Placebo Analgesia Reduces Costly Prosocial Helping to Lower Another Person’s Pain by Helena Hartmann, Paul A. G. Forbes, Markus Rütgen and Claus Lamm in Psychological Science

## Transparency

*Action Editor:* Daniela Schiller

*Editor:* Patricia J. Bauer

*Author Contributions*

**H. Hartmann:** conceptualization, data curation, formal analysis, funding acquisition, investigation, methodology, software, visualization, writing – original draft, writing – review and editing.

**P. Forbes:** conceptualization, formal analysis, methodology, supervision, writing – original draft, writing – review and editing.

**M. Rütgen:** conceptualization, formal analysis, methodology, supervision, writing – original draft, writing – review and editing.

**C. Lamm:** conceptualization, formal analysis, funding acquisition, methodology, project administration, resources, supervision, writing – original draft, writing – review and editing
