# Supplementary material for: Placebo Analgesia Reduces Costly Prosocial Helping to Lower Another Person’s Pain
Source: Psychol Sci. 2022 Sep 29;33(11):1867–81. doi: 10.1177/09567976221119727 (PMC13020941; doi:10.1177/09567976221119727)
Supplement: sj-docx-1-pss-10.1177_09567976221119727 – Supplemental material for Placebo Analgesia Reduces Costly Prosocial Helping to Lower Another Person’s Pain [file sj-docx-1-pss-10.1177_09567976221119727.docx]

Supplementary Material

**Placebo analgesia reduces costly prosocial helping to lower another’s pain**

# Procedure

Eligible participants were sent trait questionnaires to be completed online prior to coming to the lab. They were asked to abstain from alcohol, drugs, and any medication intake (except oral contraceptives) 24 hours before the experiment and to abstain from smoking, food, caffeine, and any drinks other than water one hour before the experiment. This was done to increase homogeneity of testing conditions.

During the **role assignment** procedure, the participant and the confederate were positioned on either side of a door and simultaneously each drew a ball from a box. Importantly, the two participants never met face to face, but arrived (and supposedly left) at staggered times. Both participants wore gloves during the procedure to conceal each other’s identities (e.g., age, race, etc.). They were further instructed not to speak and direct their gaze away from the box when drawing their ball. To keep up the cover story, both were asked to wave to each other once, otherwise they did not interact with each other. Since this study investigated prosocial behavior, the real participant was always be assigned the role of “Decider” and the confederate always the role of “Receiver”, independent of the colour of the ball they drew.

In the **pain calibration**, we gave short-lasting electrical stimulations in multiple trials, moving up stepwise from a rating of 0 = 'not perceivable' until the stimulus was rated as 8 = 'extremely painful but bearable' (beginning at a low intensity of 0.05 mA, step size individually chosen for each participant, so that each rating was experienced at least once). This was then repeated a second time with a small break in between to avoid habituation/sensitization and followed by varying (seemingly “random”) stimulation in the before calibrated rating range from 1 to 8. On average, input intensities for the first-hand and empathy for pain task were 0.77 ± 1.15 ($M\pm SD$) mA (range from 0.09 – 9.00 mA) for painful and 0.10 ± 0.07 mA (range from 0.01 – 0.50 mA) for non-painful stimulation.

In the **effort calibration**, participants gripped the hand dynamometer two times in a row for three seconds, while seeing live feedback of their exerted effort on the screen. They were instructed to hold the device in a certain way (lower arm and hand on a cushion on the table, dorsum of the hand directed towards the table) and asked to maintain this position during all tasks involving the grip force device. This was done to keep the MVC constant (as it can be affected by different holding positions of the dynamometer). During the first calibration, the participants were watched by an experimenter to check for correct execution and corrected if needed, e.g., to press stronger next time or hold the device differently. Participants were urged to press as strongly as they could each time. As part of the three effort calibrations and to make sure that the effort levels (effort levels 1-5 corresponding to 30, 40, 50, 60 and 70 % of their MVC, respectively) were perceived similarly in both groups, and that this perception did not change over time, participants completed two items from the NASA Task Load Index (Hart & Staveland, 1988; Effort: “How hard did you have to work to accomplish your level of performance?”; Physical Demand: “How physically demanding was the task?”) and one additional question (Unpleasantness: “How unpleasant was it for you?”) for each of the five effort levels (on a scale from 1 = not at all to 20 = extremely effortful/physically demanding/unpleasant).

In the **belief ratings** measured only in the placebo group, we asked “How effective do you currently consider the pain medication to be in reducing your pain later during the tasks?”. The Positive and Negative Affect Schedule (PANAS; Krohne et al., 1996) included 10 adjectives each for positive and negative mood, answered on a scale from 1 = Little or not at all to 5 = Extremely.

The placebo **conditioning** procedure involved the participants receiving electrical stimulation on the same hand using the exact electrode location as during calibration. These conditioning trials were repeated for a minimum of two and up to a maximum of four times until the subject did not respond with values higher than 5 to the conditioning stimulus, i.e., until it was believable that a pain reduction had taken place. In fact, the pill was a placebo and did not contain any active analgesic or pain-reducing components, but a natural sugar-alcohol (Mannitolum, 40G per pill). The control group did not receive a pill or any type of induction but had waiting times throughout the experiment to keep the session length constant between the two groups. To measure the strength of the placebo effect, we evaluated the participants’ belief in the effectiveness of the medication at three time points during the session: directly after the placebo administration (= pre-conditioning), after the placebo induction procedure (= post-conditioning) and after the completion of all tasks (= post-session). Each time, participants indicated how effective they believed the pill to be in reducing their pain in the tasks on a continuous visual analogue scale (VAS) from 0 = not effective at all to 100 = extremely effective. We then used the first-hand pain ratings in the first-hand and empathy for pain task as a main indication for the placebo effect. Finally, in the post-experimental questionnaires, we asked participants how painful the electrical stimulation had felt in the first-hand and empathy for pain task, to assess whether the belief in the pill lasted until the end of the session.

During all **tasks**, the cover story was kept up by interacting equally with the participant and the confederate, e.g., by coordinating the simultaneous starting of the tasks between the two experimenters and “waiting for the others to be ready” before continuing with the next step. Participants completed all tasks sitting in a room separated from the confederate. All participants completed a prosocial effort task and a first-hand and empathy for pain task on a computer in a fixed order. Afterwards, participants completed a third task unrelated to this project. To keep anonymity, participants were told that they arrived a few minutes apart from each other, saw only each other’s gloved hands during the role assignment and left separately at different time points.

In the **prosocial effort task**, all trials were the same length independent of the choices the participant made. Participants completed three blocks of 25 trials each, with breaks of 60s in between. Blocks of the prosocial effort task were pseudorandomized, but trials within a block were kept constant over all participants. All participants exerted the effort with their right hand and made the choices with two fingers of their left hand placed on a keyboard. As part of the cover story, participants were told that the shocks the other person received because of their choices were always “very painful”.

The **first-hand and empathy for pain task** was included as a manipulation check for evaluating analgesic effects on first-hand and empathy for pain, to then measure effects of this manipulation on prosocial behavior. Our task differed from the one used in Rütgen, Seidel, Silani, et al. (2015) in three aspects: (1) We included 20 instead of 60 trials, but collected the same amount of ratings; (2) We did not show pictures of the confederate’s face in pain as this would have interfered with the anonymity of participants; (3) we displayed “SHE"/"HE” or “YOU” in big letters instead of arrows as cues to reduce ambiguity about the target of stimulation. However, since this task was completed relatively late in the experiment in relation to the placebo induction, placebo effects could have been reduced compared to the beginning of the experiment. To counteract this, we assessed analgesic effects on empathy for pain at two time points, before (pre-effort) and after (post-effort) the prosocial effort task and used a thorough procedure for identifying non-responders to the manipulation. Twenty trials of the first-hand and empathy for pain task were shown in a pseudorandomized order (four sequences were created manually for each task and alternated over all participants in an a-priori defined order, however, the pre-effort **empathy for pain task** involved only four trials of other-related electrical stimulation (2x pain, 2x no pain) and the first-hand and empathy for pain task started with four non-random other-related stimuli and was followed by the rest of the conditions in the previously specified pseudorandom order). The ratings targeted two different aspects of empathy: (1) Rating of the own subjective degree of unpleasantness when seeing another person in pain (‘other unpleasantness’), tapping into affective sharing and vicarious distress; and (2) rating of the intensity of the pain felt by the other (‘other pain’) as a more cognitive-evaluative aspect of empathy (Lamm & Majdandžić, 2015). For first-hand stimulation, we asked participants to evaluate: 'How painful was this stimulation for you?'; for stimulation of the other person, we asked: 'How unpleasant did it feel for you when the other person was stimulated?' as well as 'How painful was this stimulation for the other person?' (all ratings were given on a 9-point rating scale from 0 = ‘not noticeable’ to 8 = ‘extremely painful/unpleasant’). In the empathy condition, the two subjective rating questions regarding pain and unpleasantness were presented in a truly random order for each trial and each participant.

The session ended with **follow-up questions**, where we also assessed any doubts that participants had regarding the study setup. Participants answered the questions about their anonymity (“My anonymity in relation to the other participant was kept”) and secrecy of decisions (“My decisions were kept secret from the other participant”) on a 5-point Likert scale from 1 = Do not agree at all to 5 = Agree completely, i.e. higher values meaning higher agreement with the statement. The monitoring question (“How often did you have the feeling that your decisions were generally ‘monitored’?”) was answered on a 5-point Likert scale from 1 = never to 5 = very often, with higher values indicating higher frequency. The whole experiment lasted approximately three hours. Participants were told that they would receive a compensation of 10€ per hour plus a bonus in one of the tasks for their participation. In reality, everybody received a total compensation of 35€. A pilot study with nine subjects was conducted before the start of data collection and prior to the upload of the preregistration to test the study procedure, especially for the placebo group. This pilot data was not used in the final analysis and is thus not reported in the present manuscript.

# Participants

Each participant was screened for exclusion criteria using an online questionnaire hosted on SoSciSurvey (Leiner, 2019), with the link sent via email. **Exclusion criteria** were a past or present enrolment in academic studies including psychology, pharmaceutics or medicine (veterinary, dental, human, etc.) as well as other medical-related trainings (e.g. nurse), any neurological or psychiatric conditions, past or present substance abuse (alcohol daily use, drugs weekly or daily use), the intake of psychopharmacological medication (besides oral contraceptives) within the last three months, and past participation in at least one placebo study or a study involving similar deceptive elements. Furthermore, participants above the clinical cut-offs of the following questionnaires were excluded: Beck’s Depression Inventory II (BDI-II; cut-off = 14; Kühner et al., 2007); Autism Quotient (AQ-k; cut-off = 17; Freitag et al., 2015); Toronto Alexithymia Scale (TAS-20; cut-off = 51; Bach et al., 1996; Table S1). Once deemed eligible, participants were pseudo-randomly allocated to either the placebo or control group. A document with subject codes and group allocation was created beforehand, where placebo and control sessions were alternated (01 = control, 02 = placebo, 03 = control, etc.). Participants with choices reflecting disengagement from the prosocial effort task, like choosing the baseline rest option in 100% of all trials (i.e., never exerting effort), having more than 10% of fail trials or failing to exert effort due to fatigue after some time, were excluded.

Regarding the **non-responder criteria**, if a participant expressed doubts about the cover story, we asked them to elaborate these further and participants with strong doubts were excluded. Exceptionally low total belief scores (sum of pre- and post-conditioning scores lower than 66.6, values can range from 0 to 200) and strong decreases between first and second measure (pre- minus post-conditioning score bigger than 33.3) served as a second measure for lack of responding. Third, we took the number of placebo conditioning trials into account. If participants responded with a value greater than 5 on a 9-point rating scale from 0 = ‘not noticeable’ to 8 = ‘extremely painful, but bearable’ to the conditioning stimulus (delivered at a medium intensity of 4), we deemed the conditioning trial as non-successful, waited another few minutes “for the medication to take effect”, and then tried again. This was repeated for a maximum of four times or until participants responded with values below 6 to the conditioning stimulus in a trial. If more than three of these trials were necessary, this was taken as an indication of non-responding to the placebo treatment and the participant was excluded from further analysis. Included participants needed an average of $M\pm SD=$2,29 ± 0.46 (range = 2-3) conditioning trials to respond to the placebo induction and no participant was excluded based on this criterion.

In total, we recruited 112 participants, nine of which were excluded for having doubts about the second participant (six in the control group, three in the placebo group), eight because of non-responding to the placebo manipulation (six because of doubts, two because of belief in the medication effectiveness), four because of > 10% of fail trials in the prosocial effort task, and one participant for not performing that task properly. This added up to ~12% of non-responders in the placebo group, which we considered comparable to the range of 8-10% non-responders in our previous work (Rütgen, Seidel, Silani, et al., 2015). This also demonstrated that all participants included in the final dataset believed the cover story.

| Table S1  Sociodemographic characteristics and trait questionnaire scores of the two groups. | | | | |
| --- | --- | --- | --- | --- |
|  | Placebo group | Control group | $t$*(*$df$*)* | $p$ |
| N (male/female)* | 45 (21/24) | 45 (21/24) | --- | --- |
| Age* | 23.56 ± 2.90 | 24.00 ± 4.32 | 0.57(76.95) | .569 |
| Empathic concern (IRI)* | 18.47 ± 4.81 | 18.40 ± 4.76 | -0.07(87.99) | .948 |
| Perspective taking (IRI)* | 18.78 ± 4.79 | 18.36 ± 3.99 | -0.45(85.21) | .651 |
| Prosocial behavior (HAS)* | 78.93 ± 8.90 | 78.82 ± 9.59 | -0.06(87.50) | .955 |
| Behavioral activation (AMI)* | 2.60 ± 0.59 | 2.52 ± 0.68 | -0.58(86.71) | .566 |
| Social motivation (AMI)* | 2.87 ± 0.61 | 2.66 ± 0.71 | -1.46(86.12) | .149 |
| Emotional sensitivity (AMI)* | 2.54 ± 0.63 | 2.61 ± 0.64 | 0.53(87.96) | .599 |
| Social Value Orientation (SVO)* | Individualists: *n* = 10  Prosocials: *n* = 35  31.95 ± 10.91 | Individualists: *n* = 7  Prosocials: *n* = 38  32.52 ± 10.65 | 0.25(87.95) | .803 |
| Psychopathy (SD3)* | 18.13 ± 3.89 | 19.62 ± 5.34 | 1.51(80.42) | .134 |
| Alexithymia (TAS-20)^+^ | 38.09 ± 6.76 | 39.93 ± 6.87 | 1.28(87.98) | .203 |
| Autism (AQ-k)^+^ | 6.62 ± 3.74 | 6.60 ± 2.99 | -0.03(83.96) | .975 |
| Depression (BDI-II)^+^ | 4.24 ± 3.67 | 4.55 ± 4.07 | 0.381(87.09) | .704 |
| *Note.* Data are given as group means or sums ± standard deviations. Group comparisons were done using Welch’s two-sample $t$-tests with group as a between-subjects factor. IRI = Interpersonal Reactivity Index (Davis, 1980; sum); HAS = Helping Attitudes Scale (Nickell, 1998; sum); AMI = Apathy Motivation Index (Ang et al., 2017, average); SVO = Social Value Orientation (reported value calculated as the arc tangent of the ratio for other- vs. self-related payoffs as an angle in degrees; Murphy et al., 2011); SD3 = Short Dark Triad (Jones & Paulhus, 2014; sum); TAS = Toronto Alexithymia Scale (Bagby et al., 1994; total sum); AQ-k = Autism Quotient Short Form (Freitag et al., 2015; sum); BDI-II = Beck Depression Inventory (Kühner et al., 2007; sum). All questionnaires were given in their original German version or were translated to German by the authors. * Scores used for matching the two groups; + Scores used for participant exclusion based on cut-offs (BDI-II cut-off = 14, AQ-k cut-off = 17, TAS-20 cut-off = 51). | | | | |

# Deviations from the preregistration

We made a mistake in describing the ANOVA with the unpleasantness ratings in the initial preregistration (<https://osf.io/g3acp>), which was corrected in an amendment (<https://osf.io/qw3kg>) uploaded during data collection. We had initially preregistered to analyze the force data with an ANOVA (just as the choice and reaction time data), but this was not possible with the ezANOVA function due to missing cells in the design (people did not always choose to exert effort in each trial and thus did not have data for every effort and/or shock reduction level). We therefore calculated an ANOVA of a linear mixed model instead. Moreover, as the two groups were comparable regarding social value orientation and apathy motivation, we refrained from repeating the main analyses of the prosocial effort task (choice, reaction time and force) with social value orientation or apathy motivation as a covariate. Lastly, we did not include computational modelling of the choice data in this paper (these analyses were mentioned as exploratory in our preregistration).

# Analyses and results

## Data analysis

Cohen’s d’s were calculated using the effect size calculation spreadsheet (version 4.2) provided by Lakens (2013). For analyses of variance (ANOVAs), we used the function ezANOVA from the R package ez, for Welch’s two-sample $t$-tests, we used the function t.test from the R package stats, for linear mixed models (LMMs), we used the function lmer (or glmer for the binary choice data) from the R package lme4 and type-III ANOVAs (Figner et al., 2020; but see Venables, 1998). Follow-up paired comparisons were computed using the Tukey method with the function pairs from the R package emmeans. Spearman’s rank correlations were computed using the function cor.test from the R package stats.

## NASA ratings

We compared the NASA ratings for effort, unpleasantness, and physical demand (manipulation check 1) of the baseline (no effort exertion, but just holding the grip force device) and the five effort levels using three ANOVAs with the factors *effort level* (1-6), *time* (pre-induction/post-induction/post-session) and *group* (placebo/control). Participants perceived the effort exertion more effortful, physically demanding, and unpleasant with increasing effort level. Below we report the full results of the ANOVAs of the NASA ratings for effort (Table S2), physical demand (Table S3) and unpleasantness (Table S4) as well as the matching graphs in Figure S1. We observed main effects of effort level in all three ratings, demonstrating that participants perceived the effort exertion more effortful ($F$(5,440) = 311.53, $p$^GG^ < .001, $\hat{\eta}_{G}^{2}$ = .40), physically demanding ($F$(5,440) = 384.74, $p$^GG^ < .001, $\hat{\eta}_{G}^{2}$ = .50), and unpleasant ($F$(5,440) = 183.21, $p$^GG^ < .001, $\hat{\eta}_{G}^{2}$ = .32) with increasing effort level (baseline rest: $M\pm SEM$ = 4.26 ± 0.36; effort level 1: 6.93 ± 0.32; effort level 2: 8.26 ± 0.28; effort level 3: 10.86 ± 0.27; effort level 4: 13.26 ± 0.26; effort level 5: 15.29 ± 0.24). Tukey post hoc contrasts indicated that all three ratings of all effort levels were significantly different from each other (all $p$ < .039). Significant main effects of time in all three ratings showed that effort ($F$(2,176) = 15.25, $p$^GG^ < .001, $\hat{\eta}_{G}^{2}$ = .02), physical demand ($F$(2,176) = 5.15, $p$^GG^ < .001, $\hat{\eta}_{G}^{2}$ = .01), and unpleasantness ($F$(2,176) = 4.81, $p$^GG^ = .008, $\hat{\eta}_{G}^{2}$ = .01) were rated differently for the three time points (pre-induction, post-induction, and post-session). Tukey post hoc contrasts indicated that the pre-induction effort ratings (10.62 ± 0.25) were significantly greater compared to the effort ratings post-induction (9.33 ± 0.26, $p$ = .001) and post-session (9.48 ± 0.27, $p$ = .005). There was a trend ($p$ = .068) for lower physical demand ratings post-induction (7.45 ± 0.23) compared to post-session (8.18 ± 0.25). Moreover, the post-induction unpleasantness ratings (5.29 ± 0.22) were significantly lower compared to the unpleasantness ratings post-session (6.15 ± 0.24, $p$ = .018). No other post hoc tests were significant. Furthermore, all three analyses showed effort level x time interactions (effort: $F$(10,880) = 13.23, $p$^GG^ < .001, $\hat{\eta}_{G}^{2}$ = . 20; physical demand: $F$(10,880) = 13.47, $p$^GG^ < .001,
$\hat{\eta}_{G}^{2}$ = . 03; unpleasantness: $F$(10,880) = 5.66, $p$^GG^ < .001, $\hat{\eta}_{G}^{2}$ = . 01; right panels in Figure S1), whereby generally, the rating differences between the five effort levels increased with increasing time, independent of group. In addition, analysis of the effort ratings showed a group x time interaction, demonstrating that the control group had significantly higher effort ratings than the placebo group pre-induction (control group: 11.43 ± 0.34; placebo group: 9.82 ± 0.36; $p$ = .024), while this was neither the case post-induction (control group: 9.92 ± 0.36; placebo group: 8.74 ± 0.39; $p$ = .201) nor post-session (control group: 9.60 ± 0.38; placebo group: 9.35 ± 0.39; $p$ = .996), independent of effort level. Importantly, we did not observe any other significant effects involving the factor group, showing that the three perceptions of the baseline and the five different effort levels were generally similar for the placebo and control group with this one exception.

| *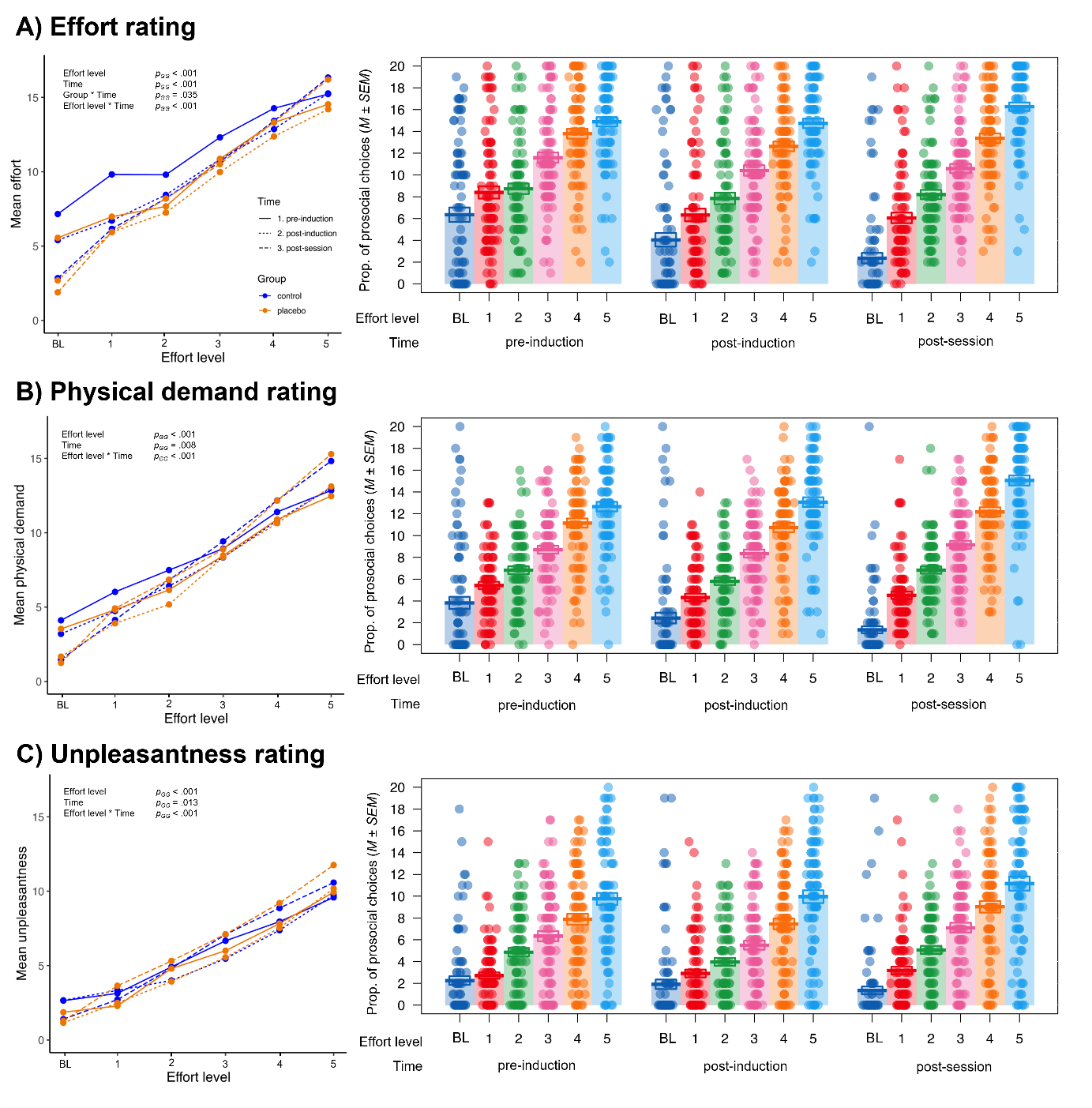* |
| --- |
| *Figure S1.* Manipulation check 1 evaluating the NASA Ratings regarding A) effort, B) physical demand and C) unpleasantness, including significant effects. Participants experienced and then rated each of the five effort levels (30, 40, 50, 60 and 70% of each participant’s maximum voluntary contraction) they also received later in the task at three time points during the session: before the placebo induction (pre-induction), after the placebo conditioning (post-induction) and post-session. GG = Greenhouse-Geisser correction; BL = baseline rest (no effort exertion). |

| Table S2  *ANOVA (Type III) of NASA effort ratings.* | | | | | | |
| --- | --- | --- | --- | --- | --- | --- |
| Effect | $\hat{\eta}_{G}^{2}$ | 90% CI | $F$ | $df$ | ${df}_{\mathrm{res}}$ | $p$ |
| Group | .012 | [.000, .075] | 1.84 | 1 | 88 | .179 |
| Effort level | .396 | [.337, .445] | 311.53 | 5 | 440 | < .001^GG^ |
| Time | .015 | [.000, .051] | 15.25 | 2 | 176 | < .001^GG^ |
| Group $\times$ Effort level | .002 | [.000, .000] | 0.98 | 5 | 440 | .389^GG^ |
| Group $\times$ Time | .004 | [.000, .022] | 3.65 | 2 | 176 | .035^GG^ |
| Effort level$\times$ Time | .022 | [.001, .029] | 13.23 | 10 | 880 | < .001^GG^ |
| Group $\times$ Effort level$\times$ Time | .002 | [.000, .000] | 1.39 | 10 | 880 | .232^GG^ |
| *Note*. $p$ values of effects including the factors effort level and/or time are reported using Greenhouse-Geisser (^GG^) correction due to the violated assumption of sphericity. | | | | | | |

| Table S3  *ANOVA (Type III) of NASA physical demand* *ratings.* | | | | | | |
| --- | --- | --- | --- | --- | --- | --- |
| Effect | $\hat{\eta}_{G}^{2}$ | 90% CI | $F$ | $df$ | ${df}_{\mathrm{res}}$ | $p$ |
| Group | .003 | [.000, .049] | 0.59 | 1 | 88 | .446 |
| Effort level | .496 | [.443, .540] | 384.74 | 5 | 440 | < .001^GG^ |
| Time | .007 | [.000, .034] | 5.15 | 2 | 176 | .008^GG^ |
| Group $\times$ Effort level | .002 | [.000, .000] | 0.66 | 5 | 440 | .533^GG^ |
| Group $\times$ Time | .002 | [.000, .016] | 1.58 | 2 | 176 | .209^GG^ |
| Effort level$\times$ Time | .027 | [.004, .036] | 13.47 | 10 | 880 | < .001^GG^ |
| Group $\times$ Effort level$\times$ Time | .002 | [.000, .000] | 1.20 | 10 | 880 | .309^GG^ |
| *Note*. $p$ values of effects including the factors effort level and/or time are reported using Greenhouse-Geisser (^GG^) correction due to the violated assumption of sphericity. | | | | | | |

| Table S4  *ANOVA (Type III) of NASA unpleasantness ratings.* | | | | | | |
| --- | --- | --- | --- | --- | --- | --- |
| Effect | $\hat{\eta}_{G}^{2}$ | 90% CI | $F$ | $df$ | ${df}_{\mathrm{res}}$ | $p$ |
| Group | .000 | [.000, .001] | 0.01 | 1 | 88 | .929 |
| Effort level | .317 | [.256, .367] | 183.21 | 5 | 440 | < .001^GG^ |
| Time | .007 | [.000, .032] | 4.81 | 2 | 176 | .013^GG^ |
| Group $\times$ Effort level | .003 | [.000, .000] | 1.05 | 5 | 440 | .349^GG^ |
| Group $\times$ Time | .002 | [.000, .014] | 1.37 | 2 | 176 | .257^GG^ |
| Effort level$\times$ Time | .008 | [.000, .008] | 5.66 | 10 | 880 | < .001^GG^ |
| Group $\times$ Effort level$\times$ Time | .001 | [.000, .000] | 0.84 | 10 | 880 | .543^GG^ |
| *Note*. $p$ values of effects including the factors effort level and/or time are reported using Greenhouse-Geisser (^GG^) correction due to the violated assumption of sphericity. | | | | | | |

## Cover story

In the analyses of the three questions about the cover story (manipulation check 2), we observed no group differences regarding participant’s perception of their anonymity in relation to the other participant ($t$(72.51) = 0.81, $p$ = .423, 95% confidence interval (CI) [-0.10, 0.23], Cohen’s $d$ = 0.17; placebo group: $M\pm SEM$ = 4.84 ± 0.07, control group: 4.91 ± 0.04), their decisions being kept secret from the other participant ($t$(87.85) = 0.36, $p$ = .717, 95% CI [-0.40, 0.57], Cohen’s $d$= 0.08; placebo group: 4.31 ± 0.18, control group: 4.40 ± 0.17), and feelings of their decisions being monitored ($t$(87.23) = 0, $p$ > .999, 95% CI [-0.55, 0.55], Cohen’s $d$ = 0.00; placebo group: 2.31 ± 0.20, control group: 2.31 ± 0.18). Generally, participants indicated high levels of kept anonymity and secrecy of their decisions. Participants of both groups expressed that they felt that their decisions were sometimes generally monitored.

## Strength, motivation, and mood

Below we report the full results of the ANOVAs analyzing the maximum voluntary contraction (Table S5) as well as changes in positive (Table S6) and negative (Table S7) mood measured at three time points during the experimental session. Figure S2 shows the analysis of the three MVCs.

| Table S5  *ANOVA (Type III) of maximum voluntary contractions over the course of the session.* | | | | | | |
| --- | --- | --- | --- | --- | --- | --- |
| Effect | $\hat{\eta}_{G}^{2}$ | 90% CI | $F$ | $df$ | ${df}_{\mathrm{res}}$ | $p$ |
| Group | .001 | [.000, .030] | 0.06 | 1 | 88 | .800 |
| Time | .001 | [.000, .002] | 0.58 | 2 | 176 | .561 |
| Group $\times$ Time | .000 | [.000, .000] | 0.31 | 2 | 176 | .736 |

| *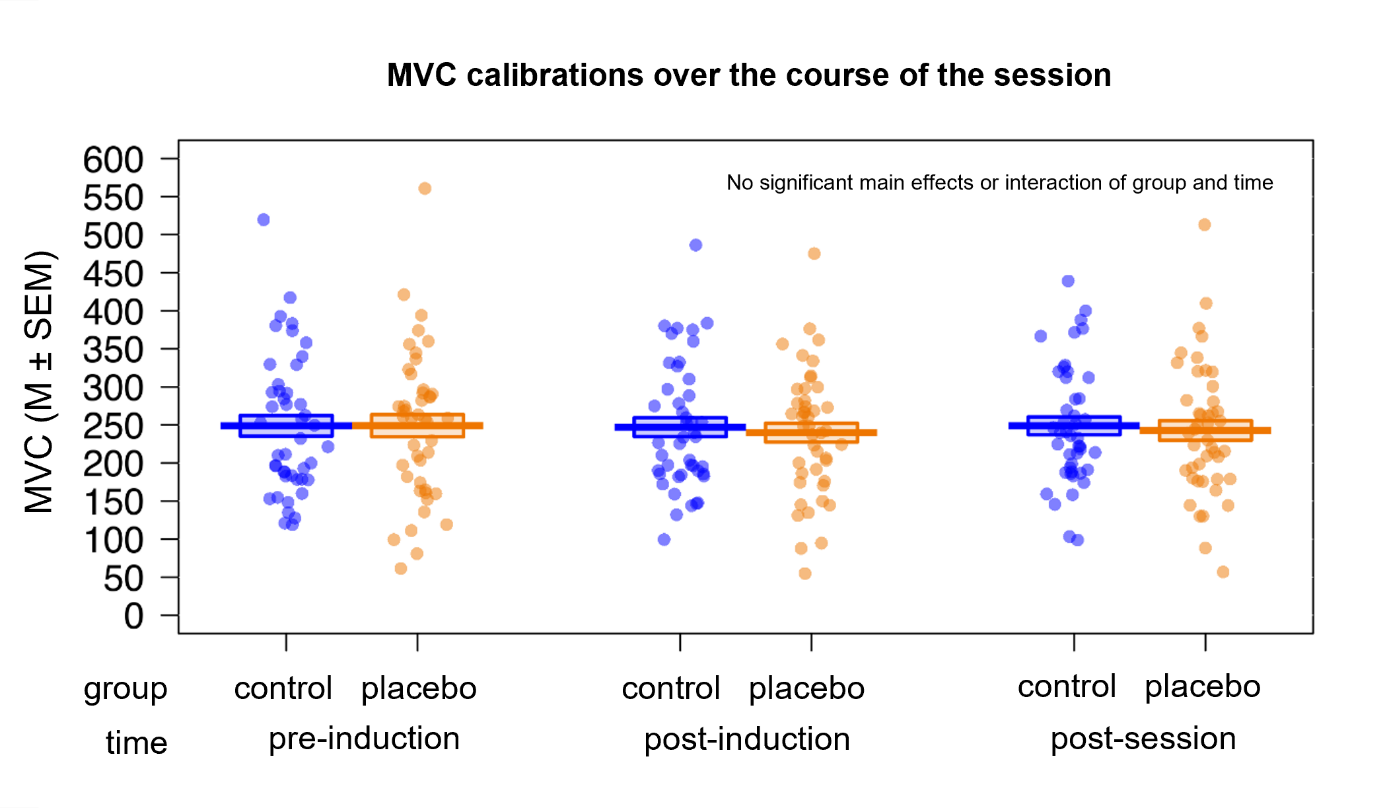* |
| --- |
| Figure S2. Post hoc analyses of maximum voluntary contraction (MVC) over the course of the session (each value was calculated as the average of two consecutive trials). We measured MVCs before (pre-induction) and after the placebo induction (post-induction) as well as at the end of the session (post-session) and compared them between the control and placebo group. |

Figure S3 shows the group comparison of the MVC (A) and force exerted (B) in the self-trial. Three subjects did not complete this task properly and thus had very low values, but exclusion of these subjects did not change the results.

| *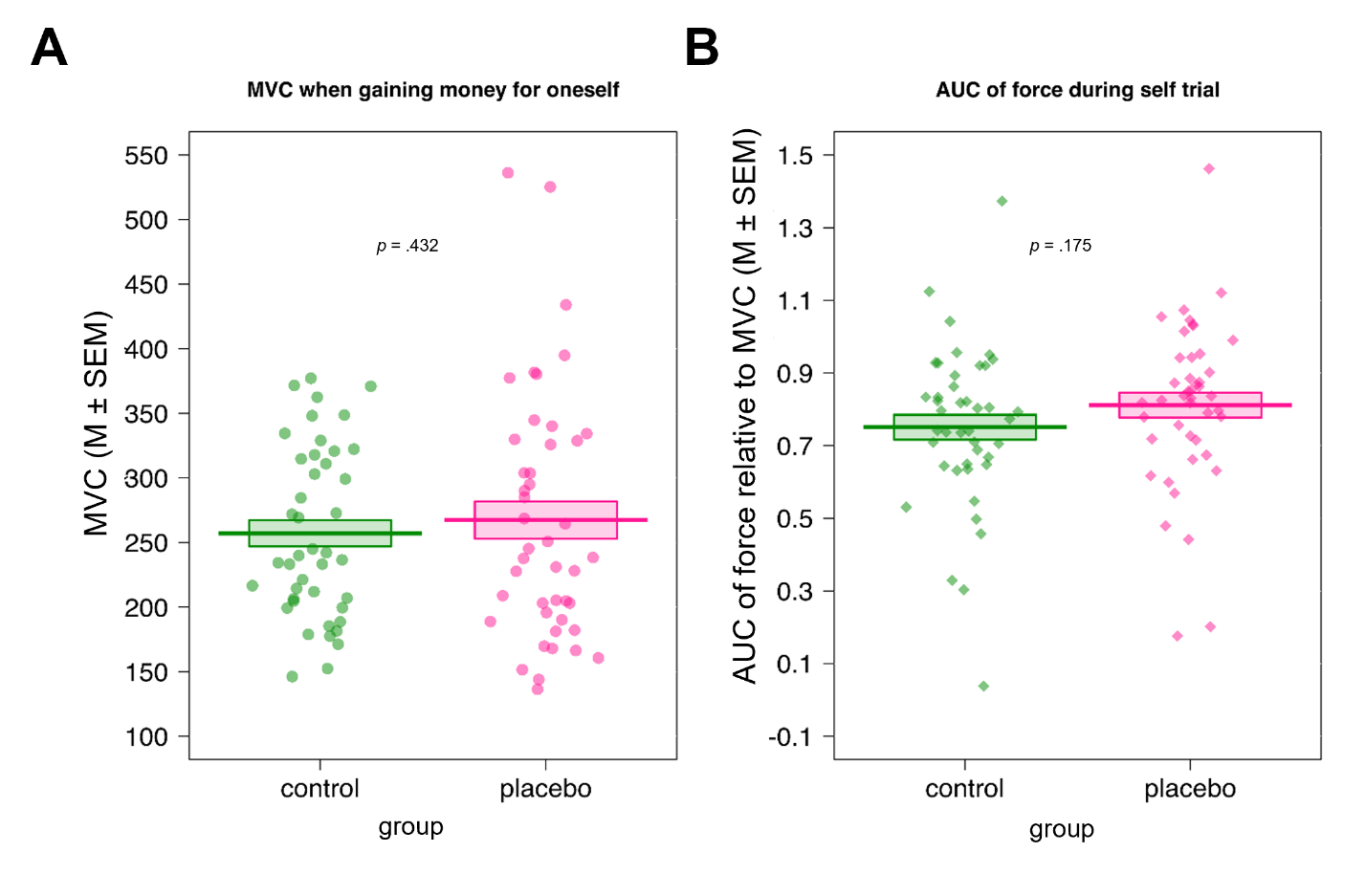* |
| --- |
| Figure S3. Preregistered analysis of the one-shot self-trial at the end of the prosocial effort task, where participants were told they could gain additional bonus money for themselves by pressing the grip force device as strongly as possible. This was operationalized once A) as the maximum voluntary contraction (MVC) and B) as the area under the curve (AUC) of the exerted force during that trial (B) and compared between the control and placebo group. Both measures showed no significant group differences, even when the three subjects, who did not complete the task properly, were excluded from the analyses. |

Figure S4 shows positive and negative mood changes over the course of the session. Regarding participants’ positive mood, we observed a main effect of time ($F$(2,176) = 43.94, $p$*^GG^* < .001, $\hat{\eta}_{G}^{2}$ = .09), showing that positive mood was lower post-session (2.56 ± 0.07) compared to pre-induction (3.01 ± 0.06, $p$ > .001) and post-induction (2.82 ± 0.07, $p$ = .010), independent of group. Evaluating the participants’ negative mood, we also observed a main effect of time ($F$(2,176) = 1.29, $p$*^GG^* < .001, $\hat{\eta}_{G}^{2}$ = .04), showing that negative mood was lower post-session (1.17 ± 0.03) compared to pre-induction (1.30 ± 0.03, $p$ = .005), again independent of group. However, negative mood was quite low in general (1.22 ± 0.02 on a scale from 1 to 5).

| Table S6  *ANOVA (Type III) of positive mood over the course of the session.* | | | | | | |
| --- | --- | --- | --- | --- | --- | --- |
| Effect | $\hat{\eta}_{G}^{2}$ | 90% CI | $F$ | $df$ | ${df}_{\mathrm{res}}$ | $p$ |
| Group | .024 | [.000, .100] | 2.69 | 1 | 88 | .104 |
| Time | .088 | [.029, .156] | 43.94 | 2 | 176 | < .001^GG^ |
| Group $\times$ Time | .005 | [.000, .012] | 2.50 | 2 | 176 | .088^GG^ |
| *Note*. $p$ values of effects including the factors effort level and/or time are reported using Greenhouse-Geisser (^GG^) correction due to the violated assumption of sphericity. | | | | | | |

| Table S7  *ANOVA (Type III) of negative mood over the course of the session.* | | | | | | |
| --- | --- | --- | --- | --- | --- | --- |
| Effect | $\hat{\eta}_{G}^{2}$ | 90% CI | $F$ | $df$ | ${df}_{\mathrm{res}}$ | $p$ |
| Group | .001 | [.000, .032] | 0.09 | 1 | 88 | .764 |
| Time | .038 | [.002, .089] | 12.92 | 2 | 176 | < .001^GG^ |
| Group $\times$ Time | .002 | [.000, .012] | 0.53 | 2 | 176 | .561^GG^ |
| *Note*. $p$ values of effects including the factors effort level and/or time are reported using Greenhouse-Geisser (^GG^) correction due to the violated assumption of sphericity. | | | | | | |

| *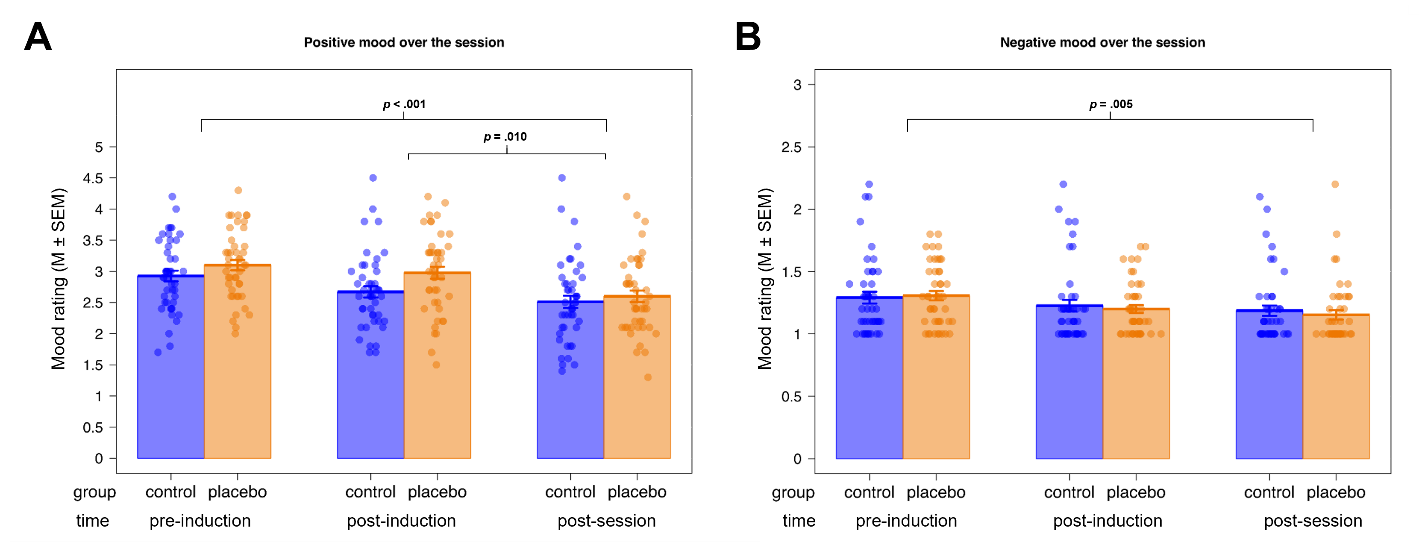* |
| --- |
| Figure S4. Post hoc analyses of A) positive and B) negative mood over the course of the session, as measured with the Positive and Negative Affect Schedule by Krohne et al. (1996). We observed main effects of time, whereby both positive and negative mood decreased over the course of the session. |

We related these control measures more directly to our prosocial task by calculating linear regressions including all control measures (self-related motivation, MVC during task, MVC change from pre- to post-conditioning, positive and negative mood changes from pre- to post- conditioning) and group predicting 1) choices, 2) reaction time, and 3) force. We found no significant effects of any of our control variables on the three outcomes (all $p$’s > .067). This suggests that apart from no group differences in our control measures, these also did not seem to be associated with any of our outcomes.

## Pre-effort empathy for pain

In the pre-effort empathy for pain task ANOVAs with either pain (Table S8) or unpleasantness (Table S9) ratings as outcomes, we only observed main effects of intensity, whereby painful stimulations the other person received were rated as more painful/unpleasant than non-painful stimulations, independent of group. No other main effects or interactions were significant in either analysis.

| Table S8  *ANOVA (Type III) of pain ratings in the pre-effort empathy for pain task.* | | | | | | |
| --- | --- | --- | --- | --- | --- | --- |
| Effect | $\hat{\eta}_{G}^{2}$ | 90% CI | $F$ | $df$ | ${df}_{\mathrm{res}}$ | $p$ |
| Group | .002 | [.000, .044] | 0.34 | 1 | 88 | .560 |
| Intensity | .739 | [.665, .792] | 671.78 | 1 | 88 | < .001 |
| Group $\times$ Intensity | .002 | [.000, .040] | 0.36 | 1 | 88 | .549 |

| Table S9  *ANOVA (Type III) of unpleasantness ratings in the pre-effort empathy for pain task.* | | | | | | |
| --- | --- | --- | --- | --- | --- | --- |
| Effect | $\hat{\eta}_{G}^{2}$ | 90% CI | $F$ | $df$ | ${df}_{\mathrm{res}}$ | $p$ |
| Group | .000 | [.000, .026] | 0.06 | 1 | 88 | .814 |
| Intensity | .409 | [.282, .516] | 230.60 | 1 | 88 | < .001 |
| Group $\times$ Intensity | .002 | [.000, .040] | 0.52 | 1 | 88 | .472 |

## Post-effort first-hand and empathy for pain task

In the post-effort first-hand and empathy for pain task ANOVA using the pain ratings as outcomes (Table S10), we observed a main effect of group ($F$(1,88) = 7.34, $p$ = .008, $\hat{\eta}_{G}^{2}$ = .81), whereby the placebo group gave lower general ratings (3.52 ± 2.40) compared to the control group (3.93 ± 2.45), independent of intensity or target. We further observed a main effect of intensity ($F$(1,88) = 1,852.53, $p$ < .001, $\hat{\eta}_{G}^{2}$ = .04), showing that, in general, painful stimulations (5.89 ± 0.09) were rated as more painful than non-painful stimulations (1.56 ± 0.07), independent of group or target. We also observed a main effect of target ($F$(1,88) = 13.94, $p$ < .001, $\hat{\eta}_{G}^{2}$ = .04), showing that other-related stimulations (3.92 ± 0.17) were rated significantly higher than self-related stimulations (3.52 ± 0.19), independent of intensity or group. Furthermore, we observed a group x target interaction ($F$(1,88) = 4.62, $p$ = .034, $\hat{\eta}_{G}^{2}$ = .01) showing that the difference between self- and other-related stimulation was bigger for the placebo (0.64 ± 0.19) compared to the control group (0.17 ± 0.11), independent of intensity. The significant intensity x target interaction ($F$(1,88) = 10.58, $p$ = .002, $\hat{\eta}_{G}^{2}$ = .01) showed that the difference between painful and non-painful stimulation was higher for self- (4.57 ± 0.14) compared to other-related stimulation (4.08 ± 0.12), independent of group. Finally, we observed a significant 3-way interaction between group, intensity, and target ($F$(1,88) = 5.61, $p$ = .020, $\hat{\eta}_{G}^{2}$ = .01), showing that the difference in the index (pain – no pain stimulation) between self- and other-related stimulation was bigger for the control (-0.85 ± 0.18) compared to the placebo group (-0.13 ± 0.24). This task was always done after the prosocial effort task.

| Table S10  *ANOVA (Type III) of pain ratings in the first-hand and empathy for pain task.* | | | | | | |
| --- | --- | --- | --- | --- | --- | --- |
| Effect | $\hat{\eta}_{G}^{2}$ | 90% CI | $F$ | $df$ | ${df}_{\mathrm{res}}$ | $p$ |
| Group | .036 | [.000, .121] | 7.34 | 1 | 88 | .008 |
| Intensity | .809 | [.753, .848] | 1,852.53 | 1 | 88 | < .001 |
| Target | .036 | [.000, .120] | 13.94 | 1 | 88 | < .001 |
| Group $\times$ Intensity | .005 | [.000, .058] | 2.36 | 1 | 88 | .128 |
| Group $\times$ Target | .012 | [.000, .076] | 4.62 | 1 | 88 | .034 |
| Intensity $\times$ Target | .013 | [.000, .079] | 10.58 | 1 | 88 | .002 |
| Group $\times$ Intensity $\times$ Target | .007 | [.000, .063] | 5.61 | 1 | 88 | .020 |

In the second ANOVA using the unpleasantness ratings (Table S11), we only observed a significant main effect of intensity ($F$(1,88) = 260.55, $p$ < .001, $\hat{\eta}_{G}^{2}$ = .40), showing that, in general, painful stimulations (4.12 ± 0.21) the other person received were rated as more unpleasant than non-painful stimulations (1.38 ± 0.13), independent of group.

| Table S11  *ANOVA (Type III) of unpleasantness ratings in the first-hand and empathy for pain task.* | | | | | | |
| --- | --- | --- | --- | --- | --- | --- |
| Effect | $\hat{\eta}_{G}^{2}$ | 90% CI | $F$ | $df$ | ${df}_{\mathrm{res}}$ | $p$ |
| Group | .009 | [.000, .069] | 1.08 | 1 | 88 | .302 |
| Intensity | .401 | [.273, .509] | 260.55 | 1 | 88 | < .001 |
| Group $\times$ Intensity | .001 | [.000, .024] | 0.21 | 1 | 88 | .648 |

## Prosocial effort task

In the prosocial effort task, participants had an $M\pm SEM$ of 0.19 ± 0.05 (12/90 subjects, range = 0-2.67) % fail trials where they did not make a decision, and 1.30 ± 0.20 (43/90 subjects, range = 0-8.00) % fail trials where they did not manage to exert the chosen effort (both relative to all trials). These fail trials did not differ significantly between the groups (decision: $t$(67.27) = -1.95, $p$ = .055, 95% confidence interval (CI) [0.09, 0.30]; squeezing: $t$(85.03) = -1.20, $p$ = .235, 95% confidence interval (CI) [1.07, 1.54]).

Below we report the full results of the three main analyses of the prosocial effort task data (Tables S12 and S13: ANOVA and LMM with proportion of work offers chosen; Tables S14 and S15: ANOVA and LMM with reaction time (RT) when making the choice; Table S16: LMM of the area under the curve of the exerted force).

In the ANOVA analyzing the proportion of work offers chosen we found a main effect of effort level ($F$(4,352) = 60.06, $p$^GG^ < .001, $\hat{\eta}_{G}^{2}$ = .13), showing that participants chose the work option less often the more effort they were asked to exert, and this was independent of the shock reduction for the other and the group (effort level 1: 0.97 ± 0.01; effort level 2: 0.95 ± 0.01; effort level 3: 0.90 ± 0.01; effort level 4: 0.80 ± 0.02; effort level 5: 0.69 ± 0.02). We also observed a main effect of shock reduction ($F$(4,352) = 63.12, $p$ < .001^GG^, $\hat{\eta}_{G}^{2}$ = .09), showing that participants chose the work option more often the more shocks they could prevent for the other person, independent of the effort level or group (preventing 5 shocks: 0.94 ± 0.01; preventing 4 shocks: 0.91 ± 0.01; preventing 3 shocks: 0.89 ± 0.01; preventing 2 shocks: 0.84 ± 0.02; preventing 1 shock: 0.71 ± 0.02).

| Table S12  *ANOVA (Type III) of the proportion of work offers chosen from the prosocial effort task.* |
| --- |

| Effect | $\hat{\eta}_{G}^{2}$ | 90% CI | $F$ | $df$ | ${df}_{\mathrm{res}}$ | $p$ |
| --- | --- | --- | --- | --- | --- | --- |
| Group | .010 | [.000, .070] | 1.96 | 1 | 88 | .165^GG^ |
| Effort level | .134 | [.077, .183] | 60.06 | 4 | 352 | < .001^GG^ |
| Shock reduction | .087 | [.039, .130] | 63.12 | 4 | 352 | < .001^GG^ |
| Group $\times$ Effort level | .001 | [.000, .000] | 0.35 | 4 | 352 | .663^GG^ |
| Group $\times$ Shock reduction | .006 | [.000, .014] | 4.10 | 4 | 352 | .020^GG^ |
| Effort level $\times$ Shock reduction | .030 | [.008, .035] | 13.11 | 16 | 1,408 | < .001^GG^ |
| Group $\times$ Effort level $\times$ Shock reduction | .003 | [.000, .000] | 1.20 | 16 | 1,408 | .304^GG^ |
| *Note*. Subject ID, group, effort level and shock reduction were entered into the ANOVA as factors; ^GG^ = Greenhouse Geisser sphericity correction. | | | | | | |

| Table S13  *Analysis of Deviance (Type III Wald test) on a generalized LMM of the proportion of work offers chosen in the prosocial effort task.* |
| --- |

| Effect | ${}^{2}$ | $df$ | $p$ |
| --- | --- | --- | --- |
| Group | 0.26 | 1 | .608 |
| Effort level | 39.78 | 4 | < .001 |
| Shock reduction | 4.83 | 4 | .305 |
| Group $\times$ Effort level | 4.72 | 4 | .317 |
| Group $\times$ Shock reduction | 13.01 | 4 | .011 |
| Effort level $\times$ Shock reduction | 24.40 | 16 | .081 |
| Group $\times$ Effort level $\times$ Shock reduction | 26.71 | 16 | .045 |
| *Note*. With choice as a binary outcome variable, we entered the factors group, effort level and shock reduction into a generalized LMM as fixed effects and included a subject-level random intercept. LMM = linear mixed model; AUC = area under the curve. A type-II test produced largely the same results, except for the main effect of shock being significant (*p* < .001). | | | |

In the ANOVA analyzing the RTs (Table S14) when making a choice, we observed main effects of effort level ($F$(3,352) = 69.84, $p$ < .001^GG^, $\hat{\eta}_{G}^{2}$ = .09) showing that participants made their choices faster for lower effort levels, independent of group or shock reduction for the other (values in ms, effort level 1: 1082.40 ± 17.03; effort level 2: 1097.29 ± 18.13; effort level 3: 1194.24 ± 20.47; effort level 4: 1326.61 ± 24.11; effort level 5: 1446.51 ± 26.24). We further observed a main effect of shock reduction ($F$(4,352) = 40.20, $p$ < .001^GG^, $\hat{\eta}_{G}^{2}$ = .03), showing that participants made their choice faster the more shocks they could prevent for the other person, independent of the effort level or group (values in ms, preventing 5 shocks: 1142.20 ± 19.31; 4 shocks: 1170.39 ± 20.87; 3 shocks: 1199.12 ± 21.77; 2 shocks: 1269.87 ± 24.25; 1 shock: 1347.48 ± 24.56). Lastly, we observed an effort level x shock reduction interaction ($F$(16,1408) = 4.88, $p$ < .001^GG^, $\hat{\eta}_{G}^{2}$ = .01).

| Table S14  *ANOVA (Type III) of the reaction time when making choices in the prosocial effort task.* |
| --- |

| Effect | $\hat{\eta}_{G}^{2}$ | 90% CI | $F$ | $df$ | ${df}_{\mathrm{res}}$ | $p$ |
| --- | --- | --- | --- | --- | --- | --- |
| Group | .015 | [.000, .083] | 2.15 | 1 | 88 | .146^GG^ |
| Effort level | .094 | [.045, .138] | 69.84 | 4 | 352 | < .001^GG^ |
| Shock reduction | .027 | [.000, .052] | 40.20 | 4 | 352 | < .001^GG^ |
| Group $\times$ Effort level | .001 | [.000, .000] | 0.95 | 4 | 352 | .383^GG^ |
| Group $\times$ Shock reduction | .001 | [.000, .000] | 1.24 | 4 | 352 | .294^GG^ |
| Effort level $\times$ Shock reduction | .009 | [.000, .006] | 4.88 | 16 | 1,408 | < .001^GG^ |
| Group $\times$ Effort level $\times$ Shock reduction | .001 | [.000, .000] | 0.60 | 16 | 1,408 | .830^GG^ |
| *Note*. Subject ID, group, effort level and shock reduction were entered into the ANOVA as factors; ^GG^ = Greenhouse Geisser sphericity correction. | | | | | | |

| Table S15  *Analysis of Deviance (Type III Wald test) on a LMM of the reaction time when making choices in the prosocial effort task.* |
| --- |

| Effect | ${}^{2}$ | $df$ | $p$ |
| --- | --- | --- | --- |
| Group | 1.49 | 1 | .222 |
| Effort level | 32.36 | 1 | < .001 |
| Shock reduction | 10.36 | 1 | .001 |
| Group $\times$ Effort level | 0.04 | 1 | .837 |
| Group $\times$ Shock reduction | 1.67 | 1 | .196 |
| Effort level $\times$ Shock reduction | 0.01 | 1 | .908 |
| Group $\times$ Effort level $\times$ Shock reduction | 1.69 | 1 | .193 |
| *Note*. With reaction time as an outcome variable, we entered the factors group, effort level, and shock reduction as well as their interaction into a LMM as fixed effects. We also included a subject-level random intercept and random slopes for effort and shock reduction as well as their interaction. LMM = linear mixed model; AUC = area under the curve. A type-II test produced the same results except for an additional group main effect (*p* = .045). | | | |

| Table S16  *Analysis of Deviance (Type III Wald test) on a LMM of the AUC of the force exerted in the prosocial effort task.* |
| --- |

| Effect | ${}^{2}$ | $df$ | $p$ |
| --- | --- | --- | --- |
| Group | 5.21 | 1 | .022 |
| Effort level | 3844.73 | 4 | < .001 |
| Shock reduction | 3.97 | 4 | .410 |
| Group $\times$ Effort level | 0.89 | 4 | .925 |
| Group $\times$ Shock reduction | 1.58 | 4 | .813 |
| Effort level $\times$ Shock reduction | 77.39 | 16 | < .001 |
| Group $\times$ Effort level $\times$ Shock reduction | 8.56 | 16 | .930 |
| *Note*. With force as an outcome variable, we entered the factors group, effort level and shock reduction into a LMM as fixed effects and included a subject-level random intercept. LMM = linear mixed model; AUC = area under the curve. A type-II test produced the same results, except for the main effect of shocks being significant (*p* = .040). | | | |

We included trial number as a covariate in our three main analyses to check whether habituation processes influenced our results. Although we observed two main effects of trial number ($p$’s < .001; for reaction time and force data), including trial number as a covariate did not change the other (especially group) effects or interpretations. Furthermore, the effort task included two rest phases and was presented in one of four pseudo-random trial orders to counteract dependency of trials, habituation and fatigue. Thus, these aspects are unlikely confounds of our findings.

## Correlations

Figure S5 shows the associations between the proportion of work offers chosen in the prosocial effort task and A) unpleasantness ratings, B) empathy for pain ratings, C) choice reaction time, and D) social value orientation.

| 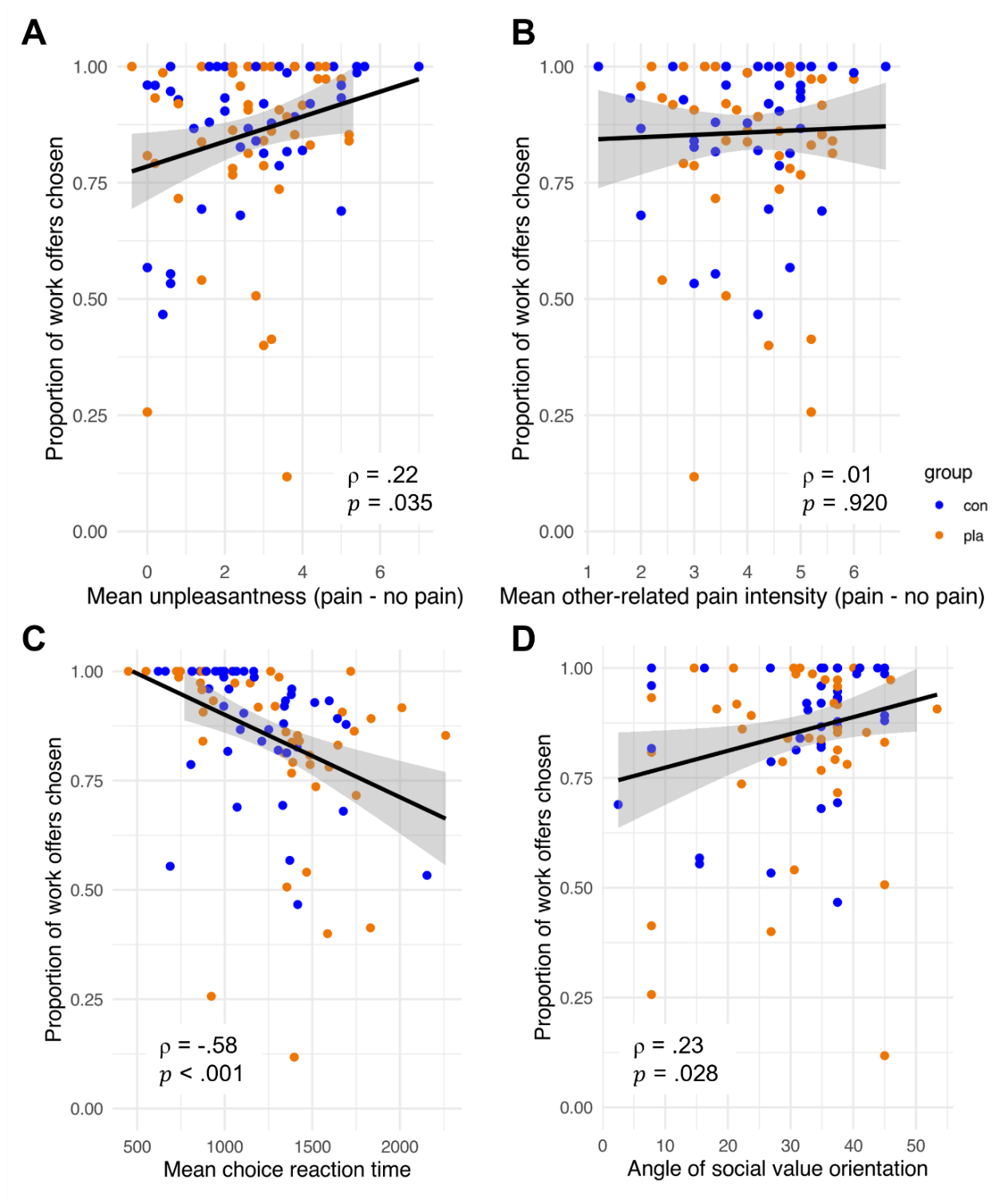 |
| --- |
| Figure S5. We computed Spearman correlations between the proportion of work offers chosen in the prosocial effort task and found A) a positive correlation with unpleasantness ratings, B) no correlation with empathy for pain ratings, C) a negative correlation with choice reaction time, and D) a positive correlation with social value orientation. All correlations were calculated over the whole sample, but color indicates group membership for display purposes: orange = control group (con); blue = placebo group (pla). We calculated correlations over the whole sample. Increased prosocial behavior in the choice task was associated to increased unpleasantness when observing others’ pain, faster reaction times when making choices and higher other-related social value orientation. |

## Mediation

Figure S6 shows the results of the mediation analysis.

| *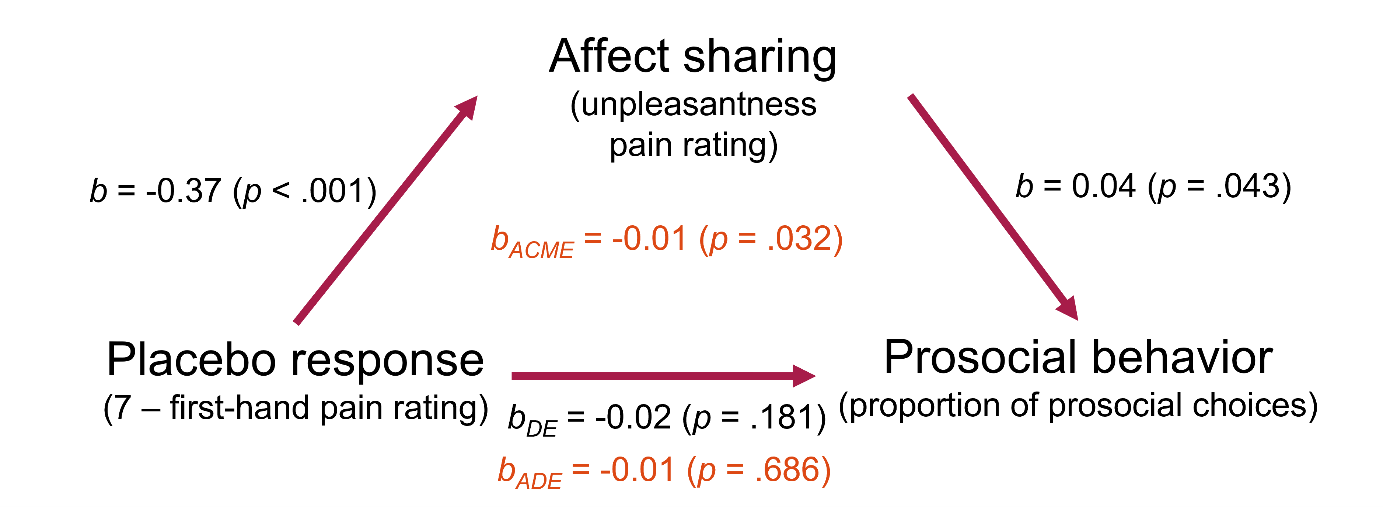* |
| --- |
| Figure S6. In the mediation analysis, the effect of placebo analgesia on prosocial choices was fully mediated via the level of affect sharing in response to others’ pain. While the (average) direct effect (b_DE_ in black and b_ADE_ in orange) between the placebo response and proportion of prosocial choices was not significant, the indirect effect (average causal mediation effect (b_ACME_ in orange); showing that the mediation effect of main interest was statistically significant. |

# References

Figner, B., Algermissen, J., Burghoorn, F., Held, L., Khalid, A., Klaassen, F., ... & Quandt, J. (2020) Standard Operating Procedures For Using Mixed-Effects Models. *A Principled Workflow from the Decision, Development, and Psychopathology (D2P2) Lab* (document version 1.0.0 -- 28 June 2020).

Venables, W. N. (1998). Exegeses on linear models. In *S-Plus User’s Conference*, Washington DC.
